# Instability of Oldroyd-B Liquid Films with Odd Viscosity on Porous Inclined Substrates

**DOI:** 10.3390/nano15030244

**Published:** 2025-02-05

**Authors:** Qingqin Zhou, Quansheng Liu, Ruigang Zhang, Zhaodong Ding

**Affiliations:** School of Mathematical Science, Inner Mongolia University, Hohhot 010021, China; zqq15247440484@163.com (Q.Z.); smslqs@imu.edu.cn (Q.L.); rgzhang@imu.edu.cn (R.Z.)

**Keywords:** Oldroyd-B fluid, liquid films, odd viscosity, porous inclined plane, instability

## Abstract

In this paper, we investigate the effect of singular viscosity on the stability of a thin film of Oldroyd-B viscoelastic fluid flowing along a porous inclined surface under the influence of a normal electric field. First, we derive the governing equations and boundary conditions for the flow of the film and assume that the film satisfies the Beavers–Joseph sliding boundary condition when it flows on a porous inclined surface. Second, through the long-wave approximation, we derive the nonlinear interfacial evolution equation. Then, linear and nonlinear stability analyses are performed for the interfacial evolution equation. The stability analyses show that the singular viscosity has a stabilizing effect on the flow of the film, while the strain delay time of the Oldroyd-B fluid, the electric field, and the parameters of the porous medium all have an unsteady effect on the flow of the film. Interestingly, in the linear stability analysis, the parameters of the porous medium have an unsteady effect on the flow of the film after a certain value is reached and a stabilizing effect before that value is reached. In order to verify these results, we performed numerical simulations of the nonlinear evolution equations using the Fourier spectral method, and the conclusions obtained are in agreement with the results of the linear stability analysis, i.e., the amplitude of the free surface decreases progressively with time in the stable region, whereas it increases progressively with time in the unstable region

## 1. Introduction

In recent years, the study of the flow stability of liquid films has become an important topic. Liquid films can be found everywhere in our daily life, industrial engineering, and scientific experiments, such as rainwater flowing down from the roof on a rainy day, latex paint on a construction site, heat exchangers, distillers, condensers, and so on, which are used in conducting scientific experiments [1,2,3,4]. Datt et al. [5] derived the thin film equations for a second-order fluid and used them to study the Landau–Levich dip-coating problem, and found that this second-order fluid model can only be used to study the stability of a thin film of a non-Newtonian fluid. When a liquid film flows on a rotating inclined plane, it may be affected by gravity, Coriolis forces, and centrifugal forces, and such instability of weakly viscoelastic films along a rotating inclined plane is relatively common in industry applications [6]. The increase in the elasticity of the viscoelastic fluid film under the influence of van der Waals forces significantly prolongs the time to film rupture [7,8]. A common non-Newtonian fluid model is the Walters’B model, which has only one non-Newtonian parameter by which the flow properties of viscoelastic fluids can be easily understood in more depth. Dholey [9] studied the stability of Walters’B fluid films flowing along inclined planes under the influence of electromagnetic fields. He found that an electric field and viscoelasticity have unstable effects on film flow, and a magnetic field has stable effects on film flow.

In addition, non-Newtonian fluids are widely used in industrial production, biomedicine, geology, and many other fields; many existing studies have focused on non-Newtonian fluids, and one of these models is the Oldroyd-B model, named after its creator, James G. Oldroyd, who proposed a mathematical structure to account for the mechanical behavior of viscoelastic fluids, which has gained a great deal of academic attention [10]. Regarding the solution of the Oldroyd-B fluid flow problem, which is also an important topic in scientific research, Khan et al. [11] used the Fourier transform method of fractional order calculus to solve the exact solution of the Oldroyd-B fluid flow in a circular tube under the influence of a magnetic field, which provided a great help in subsequent research. The problem of the stability of Oldroyd-B fluid has also received extensive attention from scholars; Jia investigated the linear time instability of a viscoelastic fluid sheet in the presence of gas velocity oscillations by using the Floquet theory and discussed the instability region in detail [12]. Moatimid et al. [13,14,15] devoted themselves to studying the stability of two-layer Oldroyd-B fluid jets; they investigated the the stability of the flow under the influence of electric or magnetic fields in different coordinate systems by varying the magnitude of different parameters and observing the effect of external electric or magnetic fields, viscosity, and elasticity of the fluid itself on the stability. The fluid film flowing down along the plane is very common in our daily life; scholars have done a lot of research on this problem; they studied the flow under different external conditions, such as the fluid flow on a porous inclined surface or a heated bottom plate; they obtained the evolution equation of the film motion through the long-wave approximation method; through the evolution equation, they can analyze the stability of the flow. The introduction of the long-wave approximation method has led to significant progress in the study of thin film problems [16,17,18,19].

Avron’s work on the Hall effect showed that, when the time inversion is broken, the viscosity tensor can have a non-vanishing singular part, at which point the liquid is endowed with a second coefficient of viscosity in addition to the one we know of, referred to as the odd viscosity or Hall viscosity [20]. As scientific research continued, Fruchart [21] also introduced odd viscosity from the point of view of continuous media, focusing on its rich phenomenology, including transverse response, mode dislocation dynamics, and topological waves. Breaking the equilibrium in the fluid through odd viscous stresses has profound implications for the dynamics of surface waves, and Doak et al. [22] explored weak and strong nonlinear waves in three-dimensional fluids with vertical odd viscosities, and their odd viscosities in this structure induced previously unexplored nonlinear effects in shallow-water waves due to surface stresses and stress gradients in the body, which made an oceanic and atmospheric geophysical contribution to the study of ocean and atmospheric geophysics. As the study of odd viscosity has progressed, it has become necessary to extend the traditional equations of kinematics in order to describe the additional hydrodynamic effects due to odd viscosity, so that it is possible to simulate the collective behavior of many particles suspended in reactive fluid media with even and odd viscosities [23]. Kirkinis et al. investigated the effect of odd viscosity on a thin viscous film of liquid on a solid substrate to support a nonlinear effect driven by the liquid–gas nonlinear capillary waves driven by surface shear stresses at the interface and found that the addition of odd viscosity inhibits the flow from becoming unstable [24]. In addition, the stability of thin films under the influence of singular viscosity when flowing in an extended inclined plane has also been considered by scholars who varied different external conditions, such as considering how the flow of the fluid changes when an external electric field is applied, when the system transfers heat, or when the inclined plane is a flexible substrate [25,26,27,28]. Interestingly, most of these studies consider Newtonian fluids, and there are fewer studies on common non-Newtonian fluids in life. Zhao et al. [29] investigated the stability of a Walters’B fluid flowing in an extended inclined plane under the influence of an odd viscosity, and found that the incorporation of the odd viscosity made the fluid more stable through the study of the nonlinear evolution equation. However, no attention has been paid to the stability of Oldroyd-B fluids under the influence of odd viscosity.

Fluid flow in porous media plays a vital role in various practical applications such as geophysics, biomedicine, petrochemicals, etc., which has led to many theoretical and experimental studies of fluid flow in porous media, and most of the literature is based on Darcy’s model or Brinkman’s model of porous media flow in different configurations [30,31,32,33]. De Oliveira et al. [34] investigated turbulent flows in porous media, illustrating the locally highly accretive nature of the gradient solution of the stabilized version of the problem. In addition, other scholars have studied the flow in porous media under different external conditions, such as electroosmotic flow, rotating channel flow, and external heating, and analyzed the influence of porous media parameters on the stability of the flow [35,36,37]. Beavers et al. [38] experimentally proposed the Beavers–Joseph slip boundary condition, which means that, when the fluid flows over the boundary layer of a porous medium, the influence of the boundary layer is replaced by a slip velocity proportional to the external velocity gradient. This provides a great convenience for us to study the flow in porous media. Li et al. investigated the instability of an odd-viscosity liquid film flowing along a porous inclined surface under the action of an applied electric field by employing the Beavers–Joseph slip boundary condition to approximate the porous wall surface, and numerically applied the fast Fourier transform method to the nonlinear evolution equation. Simulations reveal that the permeability of the porous medium has an unsteady effect on the flow of the fluid [39]. The fast Fourier transform method is a commonly used numerical simulation algorithm, which is often used in the process of solving numerical solutions of differential equations [40,41]. In addition, other scholars have used the fast Fourier transform to study problems in fluid dynamics, such as calculating the permeability of porous media, characterizing the spectral characteristics of ocean wind and waves, and so on [42,43,44]. In addition, Khan et al. investigated mixed convection in gravity-driven non-Newtonian nanofluidic films containing nanoparticles and rotating microorganisms flowing along a convection-heated vertical surface. The authors solved the problem using homogenization analysis and illustrated the effect of each parameter in the paper on the flow through the liquid film [45]. The stability of Oldroyd-B fluid under the influence of odd viscosity on a porous wall has not been studied yet.

This paper focuses on the stability of the viscoelastic liquid film of Oldroyd-B fluid with singular viscosity when it flows along the inclined porous wall under the action of an applied electric field, which helps to improve the theoretical system of non-Newtonian fluid dynamics, deepen the understanding of the stability of fluid flow, and push forward the development of related theories. First, the constitutive equations of the Oldroyd-B fluid are linearized so as to derive the continuity equation under the influence of odd viscosity and the equations of motion of the fluid flow. Then, unlike the conventional Darcy model, we use the Beavers–Joseph slip boundary condition to approximate the porous wall. Secondly, we derive the nonlinear evolution equations for a viscoelastic liquid film of odd-viscosity Oldroyd-B fluid flowing along an inclined porous wall under the influence of an applied electric field by means of a long-wave approximation. On this basis, we conducted linear and nonlinear stability studies based on the nonlinear evolution equations, and derived the effects of various physical parameters on the flow stability of the film, such as the hysteresis time of the Oldroyd-B fluid, the odd viscosity, the strength of the electric field, and porous medium parameters. Finally, we also carried out numerical simulations of the nonlinear evolution equations using the fast Fourier transform method, and judged the influence of each physical quantity on the stability by changing the values of the physical quantities and observing the amplitude of the interfaces, and the results obtained were consistent with those of the linear stability.

## 2. Formulation of the Problem

**Figure 1:**
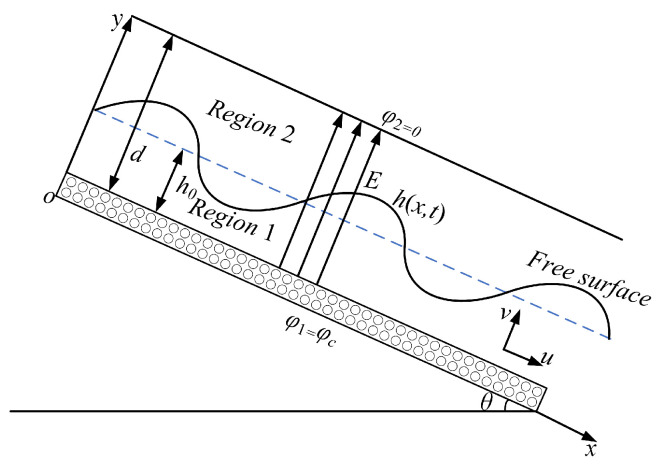
Liquid film flow down the porous inclined plane.

In this paper, we consider a two-dimensional Oldroyd-B fluid film. The film flows along an inclined porous ramp under the effect of gravity and a normal electric field. The fluid flow model diagram is shown in Figure 1. In the plane right-angle coordinate system (x,y), the *x*-axis coincides with the bottom of the plane and coincides with the flow direction, the *y*-axis is perpendicular to the porous inclined plane and points to the direction of the fluid, the inclined plane has an angle of inclination of θ, and the gravitational acceleration is *g*.

A constant potential $\varphi_{1}=\varphi_{c}$ (where $\varphi_{c}$ is a constant) is maintained at the inclined bottom plate, i.e., at y=0. We ground the top plate at y=d, i.e., $\varphi_{2}=0$. We neglect air conductivity and the magnetic field. We assume that the liquid film thickness is $h_{0}$ in the undisturbed case. In the perturbed case, the free surface of the liquid film is denoted by h(x,t).

### 2.1. Governing Equations

In Figure 1, the velocity field is denoted by V=(u,v), where *u* and *v* correspond to the flow velocity of the fluid in the horizontal and vertical directions, respectively. For an Oldroyd-B fluid, the stress tensor can be expressed as

(1) τ=−PI+S

(2) $$S+\lambda \frac{DS}{Dt}=2\eta \left(A+\Lambda \frac{DA}{Dt}\right)$$

*P* is the hydrostatic pressure, *I* is the identity tensor, *S* is an extra tensor, μ is the viscosity of the fluid, λ and Λ are constants representing the relaxation and the retardation times, respectively. $(A=\frac{1}{2}\left(\nabla V+{\left(\nabla V\right)}^{T}\right)$ is the strain-rate tensor, *V* is the velocity vector, and ∇V is the gradient operator, where

(3) $$\frac{DS}{Dt}=\left(\frac{\partial }{\partial t}+V\cdot \nabla \right)S-S\left(\nabla V\right)-{\left(\nabla V\right)}^{T}S$$

(4) $$\frac{DA}{Dt}=\left(\frac{\partial }{\partial t}+V\cdot \nabla \right)A-A\left(\nabla V\right)-{\left(\nabla V\right)}^{T}A$$

The total stress tensor is composed of two parts. The first part is the viscoelastic tensor of the Oldroyd-B model which was presented in Equations (1) and (2). In order to facilitate calculation, we ignore the nonlinear term in the expansion of the Equations (3) and (4); then, Equation (2) becomes [12,13,46]

(5) $$S=2\eta {\left(1+\lambda \frac{\partial }{\partial t}\right)}^{-1}\left(1+\Lambda \frac{\partial }{\partial t}\right)A,$$

So, the Cauchy stress tensor of Oldroyd-B fluid can be expressed as

(6) $$\begin{array}{cc} \; & \tau_{ij}=-p\delta_{ij}+\eta {\left(1+\lambda \frac{\partial }{\partial t}\right)}^{-1}\left(1+\Lambda \frac{\partial }{\partial t}\right)\left(\frac{\partial V_{i}}{\partial x_{j}}+\frac{\partial V_{j}}{\partial x_{i}}\right). \end{array}$$

where δ is the Kroneckerl symbol, defined as

(7) $$\delta_{ij}=\left\{\begin{array}{cc} 1, & i=j\\ 0, & i\neq j \end{array}\;i,j=1,2.\right.$$

It is well known that the classical Newtonian model is a special case of the Oldroyd-B model when λ=Λ=0. Furthermore, it reduces to the Maxwell model when Λ=0. When the time inversion is broken, the viscosity η in a Newtonian fluid can be written as the sum of two terms $\eta =\eta^{e}+\eta^{o}$, where $\eta^{e}$ is the even viscosity and $\eta^{o}$ is the odd viscosity [20]. Thus, from Equation (6), it can be seen that, in the expression for the stress tensor τ of the Oldroy-B fluid, the viscous stress part only has more operators than the viscous stress part of the Newtonian fluid, and the other parts are consistent with the Newtonian fluid.

In a liquid with broken time-reversal symmetry, the Cauchy stress tensor $\tau_{ij}$ consists of two parts: $\tau_{ij}=\tau_{ij}^{e}+\tau_{ij}^{o}$, where $\tau_{ij}^{e}$ and $\tau_{ij}^{o}$ are even and odd parts of the Cauchy stress tensor, respectively. For two-dimensional Oldroydian viscoelastic fluid flow of the thin film, they can be expressed as

(8) $$\tau_{ij}^{e}=-p\delta_{ij}+\eta^{e}{\left(1+\lambda \frac{\partial }{\partial t}\right)}^{-1}\left(1+\Lambda \frac{\partial }{\partial t}\right)\left(\frac{\partial V_{i}}{\partial x_{j}}+\frac{\partial V_{j}}{\partial x_{i}}\right)$$

(9) $$\begin{array}{cc} \tau_{ij}^{o}= & -\eta^{o}\left(\delta_{i1}\delta_{j1}-\delta_{i2}\delta_{j2}\right){\left(1+\lambda \frac{\partial }{\partial t}\right)}^{-1}\left(1+\Lambda \frac{\partial }{\partial t}\right)\left(\frac{\partial u}{\partial y}+\frac{\partial v}{\partial x}\right)\\ \; & +\eta^{o}\left(\delta_{i1}\delta_{j2}+\delta_{i2}\delta_{j1}\right){\left(1+\lambda \frac{\partial }{\partial t}\right)}^{-1}\left(1+\Lambda \frac{\partial }{\partial t}\right)\left(\frac{\partial u}{\partial x}-\frac{\partial v}{\partial y}\right), \end{array}$$

To sum up, the governing equation can be expressed as

(10) $$\frac{\partial u}{\partial x}+\frac{\partial v}{\partial y}=0,$$

(11) $$\rho \left(\frac{\partial u}{\partial t}+u\frac{\partial u}{\partial x}+v\frac{\partial u}{\partial y}\right)=\frac{\partial \tau_{11}}{\partial x}+\frac{\partial \tau_{12}}{\partial x}+\rho g\sin\theta ,$$

(12) $$\rho \left(\frac{\partial v}{\partial t}+u\frac{\partial v}{\partial x}+v\frac{\partial v}{\partial y}\right)=\frac{\partial \tau_{21}}{\partial x}+\frac{\partial \tau_{22}}{\partial x}-\rho g\cos\theta .$$

where ρ is the density and each component of the stress tensor is

(13) $$\begin{array}{cc} \tau_{11}= & -P+2\eta^{e}{\left(1+\lambda \frac{\partial }{\partial t}\right)}^{-1}\left(1+\Lambda \frac{\partial }{\partial t}\right)\frac{\partial u}{\partial x}-\eta^{o}{\left(1+\lambda \frac{\partial }{\partial t}\right)}^{-1}\left(1+\Lambda \frac{\partial }{\partial t}\right)\left(\frac{\partial u}{\partial y}+\frac{\partial v}{\partial x}\right) \end{array}$$

(14) $$\begin{array}{cc} \tau_{12}=\tau_{21}= & \eta^{e}{\left(1+\lambda \frac{\partial }{\partial t}\right)}^{-1}\left(1+\Lambda \frac{\partial }{\partial t}\right)\left(\frac{\partial u}{\partial y}+\frac{\partial v}{\partial x}\right)\\ \; & +\eta^{o}{\left(1+\lambda \frac{\partial }{\partial t}\right)}^{-1}\left(1+\Lambda \frac{\partial }{\partial t}\right)\left(\frac{\partial u}{\partial x}-\frac{\partial v}{\partial y}\right) \end{array}$$

(15) $$\begin{array}{cc} \tau_{22}= & -P+2\eta^{e}{\left(1+\lambda \frac{\partial }{\partial t}\right)}^{-1}\left(1+\Lambda \frac{\partial }{\partial t}\right)\frac{\partial v}{\partial y}+\eta^{o}{\left(1+\lambda \frac{\partial }{\partial t}\right)}^{-1}\left(1+\Lambda \frac{\partial }{\partial t}\right)\left(\frac{\partial u}{\partial y}+\frac{\partial v}{\partial x}\right) \end{array}$$

### 2.2. Boundary Conditions

The general solution of the equation introduced in Section 2.1 must satisfy the following nonlinear boundary conditions. For Region 2, it is considered an ideal dielectric with permittivity $\epsilon_{0}$. The electric field can be characterized by the following equation:

(16) $$E_{2}=-\nabla \varphi_{2},$$

and satisfies Gauss’s law

(17) $$\frac{\partial^{2}\varphi_{2}}{\partial x^{2}}+\frac{\partial^{2}\varphi_{2}}{\partial y^{2}}=0.$$

At the incline bottom plate y=0, we adopt the Beavers–Joseph slip boundary condition

(18) $$\left\{\begin{array}{c} \partial u/\partial y=\frac{\alpha }{\sqrt{k_{p}}}\left(u-u_{p}\right),\\ v=v_{p}, \end{array}\right.\;y=0.$$

where $k_{p}$ is the permeability of the porous medium, α is a dimensionless parameter determined by the structure of the porous medium, and $u_{p}$ and $v_{p}$ are the Dahl-averaged filtration velocities in the *x* and *y* directions, respectively [38,39]. When the pore characteristic length of the porous media is much smaller than the liquid film thickness, the Pascal scaling analysis will simplify the tilted bottom condition as

(19) $$\left\{\begin{array}{c} \partial u/\partial y=(\alpha /\sqrt{k_{p}})u,\\ v=0, \end{array}\right.\;y=0.$$

At the free surface y=h(x,t) , the conservation of mass across the interface, which is the so-called kinematic condition, yields

(20) $$v=\frac{\partial h}{\partial t}+u\frac{\partial h}{\partial x}.$$

and, together with the normal and tangential stress, balances

(21) $$n\cdot \left(\tau^{e}+\tau^{o}\right)\cdot n=-\gamma \kappa n\cdot n-p_{g}n\cdot n+n\cdot M\cdot n,$$

(22) $$n\cdot \left(\tau^{e}+\tau^{o}\right)\cdot t=0,$$

where γ is the coefficient of surface tension between the liquid and the surrounding medium, $p_{g}$ is the atmospheric pressure, n and t are the unit normal and tangent vectors y=h(x,t) at the interface pointing in the direction of the interior of region 2, and κ is the curvature of the free surface, defined as

(23) κ=∇·n.

For two-dimensional flows, the tangent and normal vectors of the free surface can be expressed as

(24) $$\begin{array}{cc} n= & \frac{\left(-\partial h/\partial x,1\right)}{\sqrt{1+{\left(\partial h/\partial x\right)}^{2}}},\\ t= & \frac{\left(1,\partial h/\partial x\right)}{\sqrt{1+{\left(\partial h/\partial x\right)}^{2}}}. \end{array}$$

The Maxwell stress tensor M is in the following form:

(25) $$M=\varepsilon_{0}E_{2}E_{2}-\frac{\varepsilon_{0}}{2}{\left|E_{2}\right|}^{2}\delta .$$

Through substitution of t, n, M, κ, $\tau^{e}$, and $\tau^{o}$ into Equations (21) and (22), we obtain

(26) $$\begin{array}{cc} \; & 2\frac{\partial h}{\partial x}{\left(1+\lambda \frac{\partial }{\partial t}\right)}^{-1}\left(1+\Lambda \frac{\partial }{\partial t}\right)\left(\frac{\partial v}{\partial y}-\frac{\partial u}{\partial x}\right)+\left[1-{\left(\frac{\partial h}{\partial x}\right)}^{2}\right]\left(\frac{\partial v}{\partial x}+\frac{\partial u}{\partial y}\right)+\eta \{[1-\\ \; & {\left(\frac{\partial h}{\partial x}\right)}^{2}]{\left(1+\lambda \frac{\partial }{\partial t}\right)}^{-1}\left(1+\Lambda \frac{\partial }{\partial t}\right)\left(\frac{\partial u}{\partial x}-\frac{\partial v}{\partial y}\right)+2\frac{\partial h}{\partial x}{\left(1+\lambda \frac{\partial }{\partial t}\right)}^{-1}\left(1+\Lambda \frac{\partial }{\partial t}\right)\\ \; & \times \left(\frac{\partial v}{\partial x}+\frac{\partial u}{\partial y}\right)\}=0, \end{array}$$

(27) $$\begin{array}{cc} \; & p_{g}-p+\frac{\eta^{e}}{1+{\left(\partial h/\partial x\right)}^{2}}\{2{\left(1+\lambda \frac{\partial }{\partial t}\right)}^{-1}\left(1+\Lambda \frac{\partial }{\partial t}\right)\frac{\partial v}{\partial y}-2\frac{\partial h}{\partial x}{\left(1+\lambda \frac{\partial }{\partial t}\right)}^{-1}\left(1+\Lambda \frac{\partial }{\partial t}\right)\\ \; & \times \left(\frac{\partial v}{\partial x}+\frac{\partial u}{\partial y}\right)+2{\left(\frac{\partial h}{\partial x}\right)}^{2}{\left(1+\lambda \frac{\partial }{\partial t}\right)}^{-1}\left(1+\Lambda \frac{\partial }{\partial t}\right)\frac{\partial u}{\partial x}+\eta [\left(1-{\left(\frac{\partial h}{\partial x}\right)}^{2}\right){\left(1+\lambda \frac{\partial }{\partial t}\right)}^{-1}\\ \; & \left(1+\Lambda \frac{\partial }{\partial t}\right)\left(\frac{\partial v}{\partial x}+\frac{\partial u}{\partial y}\right)-2\frac{\partial h}{\partial x}{\left(1+\lambda \frac{\partial }{\partial t}\right)}^{-1}\left(1+\Lambda \frac{\partial }{\partial t}\right)\left(\frac{\partial u}{\partial x}-\frac{\partial v}{\partial y}\right)]\}\\ \; & =\frac{\gamma \left(\partial^{2}h/\partial x^{2}\right)}{{\left[1+{\left(\partial h/\partial x\right)}^{2}\right]}^{3/2}}+\frac{\epsilon_{0}}{1+{\left(\partial h/\partial x\right)}^{2}}{\left(\frac{\partial h}{\partial x}\frac{\partial \varphi_{2}}{\partial x}-\frac{\partial \varphi_{2}}{\partial y}\right)}^{2}-\frac{\epsilon_{0}}{2}\left[{\left(\frac{\partial \varphi_{2}}{\partial x}\right)}^{2}+{\left(\frac{\partial \varphi_{2}}{\partial y}\right)}^{2}\right]. \end{array}$$

where η is the ratio of the odd viscosity coefficient $\eta^{o}$ to the even viscosity coefficient $\eta^{e}$ i.e., $\eta =\eta^{e}/\eta^{o}$.

### 2.3. Dimensionless Analysis

Before dealing with the numerical calculations, for more convenience, the above categories must be written in appropriate dimensionless form. First, we introduce the basic speed at the interface $u_{0}=\frac{\rho gh_{0}^{2}\sin\theta }{2\eta^{e}}$; it is the base velocity at the interface, given by the balance between the *x* component of gravity and the viscous force. $h_{0}$ denotes the unperturbed film thickness, $\varphi_{c}$ is the potential at the liquid interface, and the starred quantities are non-dimensional. The other nondimensional quantities may be given as

(28) $$\begin{array}{cc} \; & x^{*}=\frac{x}{h_{0}},y^{*}=\frac{y}{h_{0}},t^{*}=\frac{t}{h_{0}/u_{0}},\lambda^{*}=\frac{\lambda }{h_{0}/u_{0}},\Lambda^{*}=\frac{\Lambda }{h_{0}/u_{0}},\\ \; & h^{*}=\frac{h}{h_{0}},d^{*}=\frac{d}{h_{0}},u^{*}=\frac{u}{u_{0}},v^{*}=\frac{v}{u_{0}},p^{*}=\frac{p}{\eta^{e}u_{0}/h_{0}},\varphi^{*}=\frac{\varphi }{\varphi_{c}}, \end{array}$$

Using Equation (28), the governing equations can be written in dimensionless form as

(29) $$\frac{\partial u}{\partial x}+\frac{\partial v}{\partial y}=0,$$

(30) $$\begin{array}{cc} Re\left(\frac{\partial u}{\partial t}+u\frac{\partial u}{\partial x}+v\frac{\partial u}{\partial y}\right) & =-\frac{\partial p}{\partial x}+{\left(1+\lambda \frac{\partial }{\partial t}\right)}^{-1}\left(1+\Lambda \frac{\partial }{\partial t}\right)\left(\frac{\partial^{2}u}{\partial x^{2}}+\frac{\partial^{2}u}{\partial y^{2}}\right)\\ \; & -\eta {\left(1+\lambda \frac{\partial }{\partial t}\right)}^{-1}\left(1+\Lambda \frac{\partial }{\partial t}\right)\left(\frac{\partial^{2}v}{\partial x^{2}}+\frac{\partial^{2}v}{\partial y^{2}}\right)+2, \end{array}$$

(31) $$\begin{array}{cc} Re\left(\frac{\partial v}{\partial t}+u\frac{\partial v}{\partial x}+v\frac{\partial v}{\partial y}\right) & =-\frac{\partial p}{\partial y}+{\left(1+\lambda \frac{\partial }{\partial t}\right)}^{-1}\left(1+\Lambda \frac{\partial }{\partial t}\right)\left(\frac{\partial^{2}v}{\partial x^{2}}+\frac{\partial^{2}v}{\partial y^{2}}\right)\\ \; & +\eta {\left(1+\lambda \frac{\partial }{\partial t}\right)}^{-1}\left(1+\Lambda \frac{\partial }{\partial t}\right)\left(\frac{\partial^{2}u}{\partial x^{2}}+\frac{\partial^{2}u}{\partial y^{2}}\right)-2\cot\theta . \end{array}$$

In Region 2, the electrically insulating fluid justifies the stationary form of the Maxwell equations, which are reduced to the Laplace equation for the electric potential

(32) $$\frac{\partial^{2}\varphi_{2}}{\partial x^{2}}+\frac{\partial^{2}\varphi_{2}}{\partial y^{2}}=0.$$

At the free surface y=0, the normalized Beavers–Joseph slip boundary conditions of the liquid film are expressed as

(33) $$\left\{\begin{array}{c} \partial u/\partial y=u/\beta ,\\ v=0, \end{array}\;y=0,\right.$$

where $\beta =\sqrt{k_{p}}/\alpha h$ is a dimensionless parameter denoting the porous medium. It is jointly determined by the permeability of the porous medium and the structural parameters of the porous medium, and can be a good approximation of the porous inclined surface.

At the inclined top plate (y=d), we have

(34) $$\varphi_{2}=0.$$

At the free surface y=h(x,t), the normalized boundary conditions are given by

(35) $$\varphi_{2}=1,$$

(36) $$v=\frac{\partial h}{\partial t}+u\frac{\partial h}{\partial x},$$

(37) $$\begin{array}{cc} \; & 2\frac{\partial h}{\partial x}{\left(1+\lambda \frac{\partial }{\partial t}\right)}^{-1}\left(1+\Lambda \frac{\partial }{\partial t}\right)\left[\left(\frac{\partial v}{\partial y}-\frac{\partial u}{\partial x}\right)+\eta \left(\frac{\partial v}{\partial x}+\frac{\partial u}{\partial y}\right)\right]+\left[1-{\left(\frac{\partial h}{\partial x}\right)}^{2}\right]{\left(1+\lambda \frac{\partial }{\partial t}\right)}^{-1}\\ \; & \times \left(1+\Lambda \frac{\partial }{\partial t}\right)[\left(\frac{\partial v}{\partial x}+\frac{\partial u}{\partial y}\right)+\eta \left(\frac{\partial u}{\partial x}-\frac{\partial v}{\partial y})\right]=0, \end{array}$$

(38) $$\begin{array}{cc} \; & {\bar{p}}_{g}-p+\frac{1}{1+{\left(\partial h/\partial x\right)}^{2}}\{2{\left(1+\lambda \frac{\partial }{\partial t}\right)}^{-1}\left(1+\Lambda \frac{\partial }{\partial t}\right)\frac{\partial v}{\partial y}-2\frac{\partial h}{\partial x}{\left(1+\lambda \frac{\partial }{\partial t}\right)}^{-1}\left(1+\Lambda \frac{\partial }{\partial t}\right)\\ \; & \times \left(\frac{\partial v}{\partial x}+\frac{\partial u}{\partial y}\right)+2{\left(\frac{\partial h}{\partial x}\right)}^{2}{\left(1+\lambda \frac{\partial }{\partial t}\right)}^{-1}\left(1+\Lambda \frac{\partial }{\partial t}\right)\frac{\partial u}{\partial x}+\eta [\left(1-{\left(\frac{\partial h}{\partial x}\right)}^{2}\right){\left(1+\lambda \frac{\partial }{\partial t}\right)}^{-1}\\ \; & \left(1+\Lambda \frac{\partial }{\partial t}\right)\left(\frac{\partial v}{\partial x}+\frac{\partial u}{\partial y}\right)-2\frac{\partial h}{\partial x}{\left(1+\lambda \frac{\partial }{\partial t}\right)}^{-1}\left(1+\Lambda \frac{\partial }{\partial t}\right)\left(\frac{\partial u}{\partial x}-\frac{\partial v}{\partial y}\right)]\}\\ \; & =\frac{\partial^{2}h/\partial x^{2}}{Ca{\left[1+{\left(\partial h/\partial x\right)}^{2}\right]}^{3/2}}+\frac{2E}{1+{\left(\partial h/\partial x\right)}^{2}}{\left(\frac{\partial h}{\partial x}\frac{\partial \varphi_{2}}{\partial x}-\frac{\partial \varphi_{2}}{\partial y}\right)}^{2}-E\left[{\left(\frac{\partial \varphi_{2}}{\partial x}\right)}^{2}+{\left(\frac{\partial \varphi_{2}}{\partial y}\right)}^{2}\right], \end{array}$$

where

(39) $${\bar{P}}_{g}=\frac{P_{g}}{\eta^{e}u_{0}/h_{0}},$$

The dimensionless parameters are the Reynolds number Re [2,29], the capillary number Ca [39], and the electrical parameter *E* [26]. They are respectively expressed as

$$Re=\frac{\rho u_{0}h_{0}}{\eta^{e}},\;Ca=\frac{\eta^{e}u_{0}}{\gamma }=\frac{Re^{2/3}\sin^{1/3}\theta }{2^{1/3}K},\;E=\frac{\varphi_{c}^{2}\epsilon_{0}}{2\eta^{e}u_{0}h_{0}},$$

where we use the Kapitza number $K=\frac{\gamma \rho^{1/3}}{g^{1/3}\eta^{e4/3}}$ as the dimensionless ratio of surface tension to inertial force. *K* is a constant for a specific material at a specific temperature.

## 3. Nonlinear Evolution Equation

In order to obtain the nonlinear evolution equation of the film flow, we use the long-wave approximation. First, it is assumed that the unperturbed thickness of the liquid film $h_{0}$ is much smaller than the interfacial deformation wavelength κ, i.e., $h_{0}/\kappa =\delta \ll 1$, where δ is a small parameter (film parameter). Next, we conduct the following variable transformations:

(40) ξ=δx,τ=δt,y=y,v=δw.

To ensure that all models have surface tension effects, the capillary number Ca should be adjusted as

(41) $$Ca^{\prime }=Ca/\delta^{2}.$$

Finally, substituting Equation (40) into dimensionless control Equations (29)–(31), we obtain

(42) $$\frac{\partial u}{\partial \xi }+\frac{\partial w}{\partial y}=0,$$

(43) $$\begin{array}{cc} \delta Re\left(\frac{\partial u}{\partial \tau }+u\frac{\partial u}{\partial \xi }+w\frac{\partial u}{\partial y}\right)= & -\delta \frac{\partial p}{\partial \xi }+{\left(1+\delta \lambda \frac{\partial }{\partial \tau }\right)}^{-1}\left(1+\delta \Lambda \frac{\partial }{\partial \tau }\right)\left(\delta^{2}\frac{\partial^{2}u}{\partial \xi^{2}}+\frac{\partial^{2}u}{\partial y^{2}}\right)\\ \; & -\eta {\left(1+\delta \lambda \frac{\partial }{\partial \tau }\right)}^{-1}\left(1+\delta \Lambda \frac{\partial }{\partial \tau }\right)\left(\delta^{3}\frac{\partial^{2}w}{\partial \xi^{2}}+\delta \frac{\partial^{2}w}{\partial y^{2}}\right)+2, \end{array}$$

(44) $$\begin{array}{cc} \delta^{2}Re\left(\frac{\partial w}{\partial \tau }+u\frac{\partial w}{\partial \xi }+w\frac{\partial w}{\partial y}\right) & =-\frac{\partial p}{\partial y}+{\left(1+\delta \lambda \frac{\partial }{\partial \tau }\right)}^{-1}\left(1+\delta \Lambda \frac{\partial }{\partial \tau }\right)\left(\delta^{3}\frac{\partial^{2}w}{\partial \xi^{2}}+\delta \frac{\partial^{2}w}{\partial y^{2}}\right)\\ \; & +\eta {\left(1+\delta \lambda \frac{\partial }{\partial \tau }\right)}^{-1}\left(1+\delta \Lambda \frac{\partial }{\partial \tau }\right)\left(\delta^{2}\frac{\partial^{2}u}{\partial \xi^{2}}+\frac{\partial^{2}u}{\partial y^{2}}\right)-2\cot\theta . \end{array}$$

The equation and boundary conditions of the potential function $\varphi_{2}$ are

(45) $$\delta^{2}\frac{\partial^{2}\varphi_{2}}{\partial \xi^{2}}+\frac{\partial^{2}\varphi_{2}}{\partial y^{2}}=0,$$

(46) $${\left.\varphi_{2}\right|}_{y=h}=1,$$

(47) $${\left.\varphi_{2}\right|}_{y=d}=0.$$

At the incline bottom plate y=0, the Beavers–Joseph slip boundary conditions become

(48) $$\left\{\begin{array}{c} \partial u/\partial y=u/\beta ,\\ w=0, \end{array}\;y=0,\right.$$

At the free surface y=h(ξ,τ), the boundary condition changes to

(49) $$w=\frac{\partial h}{\partial \tau }+u\frac{\partial h}{\partial \xi },$$

(50) $$\begin{array}{cc} \; & 2\delta \frac{\partial h}{\partial \xi }[{\left(1+\delta \lambda \frac{\partial }{\partial \tau }\right)}^{-1}\left(1+\delta \Lambda \frac{\partial }{\partial \tau }\right)\left(\delta \frac{\partial w}{\partial y}-\delta \frac{\partial u}{\partial \xi }\right)+\eta {\left(1+\delta \lambda \frac{\partial }{\partial \tau }\right)}^{-1}\left(1+\delta \Lambda \frac{\partial }{\partial \tau }\right)(\delta^{2}\frac{\partial w}{\partial \xi }\\ \; & +\frac{\partial u}{\partial y})]+\left[1-\delta^{2}{\left(\frac{\partial h}{\partial \xi }\right)}^{2}\right][{\left(1+\delta \lambda \frac{\partial }{\partial \tau }\right)}^{-1}\left(1+\delta \Lambda \frac{\partial }{\partial \tau }\right)\left(\delta^{2}\frac{\partial w}{\partial \xi }+\frac{\partial u}{\partial y}\right)+\eta {\left(1+\delta \lambda \frac{\partial }{\partial \tau }\right)}^{-1}\\ \; & \left(1+\delta \Lambda \frac{\partial }{\partial \tau }\right)\left(\delta \frac{\partial u}{\partial \xi }-\delta \frac{\partial w}{\partial y}\right)]=0, \end{array}$$

(51) $$\begin{array}{cc} \; & {\bar{p}}_{g}-p+\frac{1}{1+\delta^{2}{\left(\partial h/\partial \xi \right)}^{2}}\{2\delta^{3}{\left(\frac{\partial h}{\partial \xi }\right)}^{2}{\left(1+\delta \lambda \frac{\partial }{\partial \tau }\right)}^{-1}\left(1+\delta \Lambda \frac{\partial }{\partial \tau }\right)\frac{\partial u}{\partial \xi }-2\delta \frac{\partial h}{\partial \xi }{\left(1+\delta \lambda \frac{\partial }{\partial \tau }\right)}^{-1}\\ \; & \left(1+\delta \Lambda \frac{\partial }{\partial \tau }\right)\left(\delta^{2}\frac{\partial w}{\partial \xi }+\frac{\partial u}{\partial y}\right)+2\delta {\left(1+\delta \lambda \frac{\partial }{\partial \tau }\right)}^{-1}\left(1+\delta \Lambda \frac{\partial }{\partial \tau }\right)\frac{\partial w}{\partial y}+\eta [\left(1-\delta^{2}{\left(\frac{\partial h}{\partial \xi }\right)}^{2}\right)\\ \; & \times {\left(1+\delta \lambda \frac{\partial }{\partial \tau }\right)}^{-1}\left(1+\delta \Lambda \frac{\partial }{\partial \tau }\right)\left(\delta^{2}\frac{\partial w}{\partial \xi }+\frac{\partial u}{\partial y}\right)-2\delta^{2}\frac{\partial h}{\partial \xi }{\left(1+\delta \lambda \frac{\partial }{\partial \tau }\right)}^{-1}\left(1+\delta \Lambda \frac{\partial }{\partial \tau }\right)\\ \; & \times \left(\frac{\partial u}{\partial \xi }-\frac{\partial w}{\partial y}\right)]\}=\frac{\partial^{2}h/\partial \xi^{2}}{Ca^{\prime }{\left[1+\delta^{2}{\left(\partial h/\partial \xi \right)}^{2}\right]}^{3/2}}+\frac{2E}{1+\delta^{2}{\left(\partial h/\partial \xi \right)}^{2}}{\left(\delta^{2}\frac{\partial h}{\partial \xi }\frac{\partial \varphi_{2}}{\partial \xi }-\frac{\partial \varphi_{2}}{\partial y}\right)}^{2}\\ \; & -E\left[\delta^{2}{\left(\frac{\partial \varphi_{2}}{\partial \xi }\right)}^{2}+{\left(\frac{\partial \varphi_{2}}{\partial y}\right)}^{2}\right]. \end{array}$$

We expand the physical quantities *u*, *w*, *p*, and $\varphi_{2}$ into the following power series forms, respectively:

(52) $$\begin{array}{cc} \; & u=u_{0}+\delta u_{1}+\cdots ,\\ \; & w=w_{0}+\delta w_{1}+\cdots ,\\ \; & p=p_{0}+\delta p_{1}+\cdots ,\\ \; & \varphi_{2}=\varphi_{20}+\delta \varphi_{21}+\cdots . \end{array}$$

In the case of ignoring O(δ) and higher order terms, we substitute the asymptotic expansion Equation (52) into the dimensionless equation, and then compare the degree of δ to obtain the zero-order governing equation

(53) $$\frac{\partial u_{0}}{\partial \xi }+\frac{\partial w_{0}}{\partial y}=0,$$

(54) $$\frac{\partial^{2}u_{0}}{\partial y^{2}}+2=0,$$

(55) $$-\frac{\partial p_{0}}{\partial y}+\eta \frac{\partial^{2}u_{0}}{\partial y^{2}}-2\cot\theta =0,$$

(56) $$\frac{\partial^{2}\varphi_{20}}{\partial y^{2}}=0.$$

At the bottom plate y=0, we obtain

(57) $$w_{0}=0,$$

(58) $$\frac{\partial u_{0}}{\partial y}=\frac{1}{\beta }u_{0},$$

At the top plate y=d, we obtain

(59) $$\varphi_{20}=0.$$

At the free surface y=h(x,t), the zero-order boundary condition is

(60) $$\frac{\partial u_{0}}{\partial y}=0,$$

(61) $${\bar{p}}_{g}-p_{0}+\eta \frac{\partial u_{0}}{\partial y}=\frac{1}{Ca^{\prime }}\frac{\partial^{2}h}{\partial \xi^{2}}+E{\left(\frac{\partial \varphi_{20}}{\partial y}\right)}^{2},$$

(62) $$\varphi_{20}=1,$$

The solution to the zero-order equation is

(63) $$u_{0}=-y^{2}+2hy+2\beta h,$$

(64) $$w_{0}=-y^{2}\frac{\partial h}{\partial \xi }-2y\beta \frac{\partial h}{\partial \xi },$$

(65) $$p_{0}=-2\left(\eta +\cot\theta \right)\left(y-h\right)-\frac{1}{Ca^{\prime }}\frac{\partial^{2}h}{\partial \xi^{2}}-\frac{E}{{\left(h-d\right)}^{2}}-{\bar{p}}_{g},$$

(66) $$\varphi_{20}=\frac{y-d}{h-d}.$$

In the same way as for the derivation of the zeroth-order equation, the asymptotic expansion Equation (52) is again substituted into the dimensionless control equation and δ is compared a number of times to obtain the first-order control equation as follows:

(67) $$\frac{\partial u_{1}}{\partial \xi }+\frac{\partial w_{1}}{\partial y}=0,$$

(68) $$\begin{array}{cc} Re\left(\frac{\partial u_{0}}{\partial \tau }+u_{0}\frac{\partial u_{0}}{\partial \xi }+w_{0}\frac{\partial u_{0}}{\partial y}\right)= & -\frac{\partial p_{0}}{\partial \xi }+\frac{\partial^{2}u_{1}}{\partial y^{2}}+\Lambda \frac{\partial }{\partial \tau }\left(\frac{\partial^{2}u_{0}}{\partial y^{2}}\right)-\eta \frac{\partial^{2}w_{0}}{\partial y^{2}}, \end{array}$$

(69) $$-\lambda \frac{\partial }{\partial \tau }\left(\frac{\partial p_{0}}{\partial y}\right)-\frac{\partial p_{1}}{\partial y}+\frac{\partial^{2}w_{0}}{\partial y^{2}}-\eta \frac{\partial^{2}u_{1}}{\partial y^{2}}+\eta \Lambda \frac{\partial }{\partial \tau }\left(\frac{\partial^{2}u_{0}}{\partial y^{2}}\right)=0.$$

At the porous bottom plate y=0, the first-order boundary condition is

(70) $$w_{1}=0,$$

(71) $$\frac{\partial u_{1}}{\partial y}=\frac{1}{\beta }u_{1},$$

At the free surface y=h(x,t), the first-order boundary condition is

(72) $$\frac{\partial u_{1}}{\partial y}+\Lambda \frac{\partial }{\partial \tau }\left(\frac{\partial u_{0}}{\partial y}\right)+\eta \frac{\partial u_{0}}{\partial \xi }-\eta \frac{\partial w_{0}}{\partial y}=0,$$

(73) $$\begin{array}{cc} \; & -\lambda \frac{\partial p_{0}}{\partial y}-p_{1}+2\frac{\partial w_{0}}{\partial y}-2\frac{\partial h}{\partial \xi }\frac{\partial u_{0}}{\partial y}+\eta \frac{\partial u_{1}}{\partial y}+\eta \Lambda \frac{\partial }{\partial \tau }\left(\frac{\partial u_{0}}{\partial y}\right)=\frac{\lambda }{Ca^{\prime }}\frac{\partial }{\partial \tau }\left(\frac{\partial^{2}h}{\partial \xi^{2}}\right)\\ \; & +\lambda E\frac{\partial }{\partial \tau }\left[{\left(\frac{\partial \varphi_{20}}{\partial y}\right)}^{2}\right]. \end{array}$$

An expression of $u_{1}$ can be obtained by solving the first-order equation

(74) $$\begin{array}{cc} u_{1}= & \left(\frac{1}{3}Rey^{3}+Re\beta y^{2}-2\Lambda y-Reh^{2}y-2Re\beta hy-2\beta \Lambda -Re\beta h^{2}-2Re\beta^{2}h\right)\frac{\partial h}{\partial \tau }\\ \; & +Re\left(\frac{\beta y^{4}}{6}\frac{\partial h}{\partial \xi }+\frac{y^{4}h}{6}\frac{\partial h}{\partial \xi }+\frac{2\beta y^{3}h}{3}\frac{\partial h}{\partial \xi }+2\beta^{2}y^{2}h\frac{\partial h}{\partial \xi }\right)+y^{2}\cot\theta \frac{\partial h}{\partial \xi }-\frac{y^{2}}{2Ca^{\prime }}\frac{\partial^{3}h}{\partial \xi^{3}}\\ \; & +\frac{Ey^{2}}{{\left(h-d\right)}^{3}}\frac{\partial h}{\partial \xi }-Re\left(\frac{8\beta yh^{3}}{3}\frac{\partial h}{\partial \xi }+\frac{2yh^{4}}{3}\frac{\partial h}{\partial \xi }+4\beta^{2}yh^{2}\frac{\partial h}{\partial \xi }\right)-2yh\cot\theta \frac{\partial h}{\partial \xi }+\frac{yh}{Ca^{\prime }}\frac{\partial^{3}h}{\partial \xi^{3}}\\ \; & -\frac{2Eyh}{{\left(h-d\right)}^{3}}\frac{\partial h}{\partial \xi }-4\eta yh\frac{\partial h}{\partial \xi }-4\eta \beta y\frac{\partial h}{\partial \xi }-Re\beta \left(\frac{8\beta h^{3}}{3}\frac{\partial h}{\partial \xi }+\frac{2h^{4}}{3}\frac{\partial h}{\partial \xi }+4\beta^{2}h^{2}\frac{\partial h}{\partial \xi }\right)\\ \; & -h\frac{\partial h}{\partial \xi }\left(2\beta \cot\theta +\frac{2E\beta }{{\left(h-d\right)}^{3}}+4\eta \beta \right)-4\eta \beta^{2}\frac{\partial h}{\partial \xi }+\frac{\beta h}{Ca^{\prime }}\frac{\partial^{3}h}{\partial \xi^{3}}. \end{array}$$

In order to obtain the nonlinear evolution equation of the thin film, we define the local flow rate q(ξ,τ), whose expression is

(75) $$q\left(\xi ,\tau \right)=\int_{0}^{h\left(\xi ,\tau \right)}u\left(\xi ,y,\tau \right)dy.$$

By substituting $u\left(\xi ,y,\tau \right)=u_{0}\left(\xi ,y,\tau \right)+\delta u_{1}\left(\xi ,y,\tau \right)+O\left(\delta^{2}\right)$ into Equation (75) and solving the integral, we obtain

(76) $$\begin{array}{cc} q\left(\xi ,\tau \right)= & \frac{2}{3}h^{3}+2\beta h^{2}+\delta [\frac{8Reh^{6}}{15}\frac{\partial h}{\partial \xi }+\frac{16Re\beta h^{5}}{5}\frac{\partial h}{\partial \xi }+\left(2\Lambda +\frac{20}{3}Re\beta^{2}\right)h^{4}\frac{\partial h}{\partial \xi }\\ \; & -\frac{2}{3}\left(-12\beta \Lambda +\cot\theta +\frac{E}{{\left(h-d\right)}^{3}}+3\eta -6Re\beta^{3}\right)h^{3}\frac{\partial h}{\partial \xi }-(-8\Lambda \beta^{2}\\ \; & +6\eta \beta +2\beta \cot\theta +\frac{2E\beta }{{\left(h-d\right)}^{3}})h^{2}\frac{\partial h}{\partial \xi }-4\eta \beta^{2}h\frac{\partial h}{\partial \xi }+\frac{h^{3}}{3Ca^{\prime }}\frac{\partial^{3}h}{\partial \xi^{3}}+\frac{\beta h^{2}}{Ca^{\prime }}\frac{\partial^{3}h}{\partial \xi^{3}}], \end{array}$$

Another form of the kinematic boundary condition can be expressed in terms of the evolution equation for the local flow rate q(ξ,τ) and the local film thickness h(ξ,τ), as follows:

(77) $$\frac{\partial h}{\partial \tau }+\frac{\partial q}{\partial \xi }=0,$$

Substituting Equation (76) into the kinematic boundary condition Equation (77) and substituting back the variables *x* and *t*, the nonlinear evolution equation for the Oldroyd-B viscoelastic film is obtained as

(78) $$\begin{array}{cc} \; & \frac{\partial h}{\partial t}+\frac{\partial }{\partial x}[\frac{2}{3}h^{3}+2\beta h^{2}+\frac{8Reh^{6}}{15}\frac{\partial h}{\partial x}+\frac{16Re\beta h^{5}}{5}\frac{\partial h}{\partial x}+\left(2\Lambda +\frac{20}{3}Re\beta^{2}\right)h^{4}\frac{\partial h}{\partial x}-\frac{2}{3}(-12\beta \Lambda \\ \; & +\cot\theta +\frac{E}{{\left(h-d\right)}^{3}}+3\eta -6Re\beta^{3})h^{3}\frac{\partial h}{\partial x}-\left(-8\Lambda \beta^{2}+6\eta \beta +2\beta \cot\theta +\frac{2E\beta }{{\left(h-d\right)}^{3}}\right)h^{2}\frac{\partial h}{\partial x}\\ \; & -4\eta \beta^{2}h\frac{\partial h}{\partial x}+\frac{h^{3}}{3Ca}\frac{\partial^{3}h}{\partial x^{3}}+\frac{\beta h^{2}}{Ca}\frac{\partial^{3}h}{\partial x^{3}}]=0. \end{array}$$

## 4. Linear Stability Analysis

In order to study the stability of the film flow, the film thickness in the perturbed state can be written as $h\left(x,t\right)=1+h_{1}\left(x,t\right)$, where $|h_{1}|\ll 1$, substituting into Equation (78), ignoring the higher-order terms of $O(h_{1})$, and keeping to $O(h_{1}^{3})$, we obtain

(79) $$\begin{array}{cc} \; & \frac{\partial h_{1}}{\partial t}+A\frac{\partial h_{1}}{\partial x}+B\frac{\partial^{2}h_{1}}{\partial x^{2}}+C\frac{\partial^{4}h_{1}}{\partial x^{4}}+A^{\prime }h_{1}\frac{\partial h_{1}}{\partial x}+B^{\prime }\left[h_{1}\frac{\partial^{2}h_{1}}{\partial x^{2}}+{\left(\frac{\partial h_{1}}{\partial x}\right)}^{2}\right]+C^{\prime }(h_{1}\frac{\partial^{4}h_{1}}{\partial x^{4}}+\frac{\partial h_{1}}{\partial x}\\ \; & \times \frac{\partial^{3}h_{1}}{\partial x^{3}})+\frac{1}{2}A^{\prime \prime }h_{1}^{2}\frac{\partial h_{1}}{\partial x}+B^{\prime \prime }\left[\frac{1}{2}h_{1}^{2}\frac{\partial^{2}h_{1}}{\partial x^{2}}+h_{1}{\left(\frac{\partial h_{1}}{\partial x}\right)}^{2}\right]+C^{\prime \prime }\left[\frac{1}{2}h_{1}^{2}\frac{\partial^{4}h_{1}}{\partial x^{4}}+h_{1}\frac{\partial h_{1}}{\partial x}\frac{\partial^{3}h_{1}}{\partial x^{3}}\right]=0, \end{array}$$

where

(80) $$A\left(h\right)=2h^{2}+4\beta h,$$

(81) $$\begin{array}{cc} B\left(h\right) & =\frac{8}{15}h^{6}Re+\frac{16}{5}h^{5}\beta Re+\left(2\Lambda +\frac{20}{3}\beta^{2}Re\right)h^{4}-\frac{2}{3}h^{3}(-12\Lambda \beta +\cot\theta +3\eta -6\beta^{3}Re\\ \; & +\frac{E}{{\left(h-d\right)}^{3}})-h^{2}\left(-8\Lambda \beta^{2}+6\eta \beta +2\beta \cot\theta +\frac{2E\beta }{{\left(h-d\right)}^{3}}\right)-4\eta \beta^{2}h, \end{array}$$

(82) $$C\left(h\right)=\frac{1}{3Ca}h^{3}+\frac{\beta }{Ca}h^{2}.$$

In Equation (79), *A*, *B*, and *C* and their corresponding derivatives denote the values at h=1. We assuming that *h* is in the form of a regular modulus, denoted as

(83) $$h_{1}=\Gamma \exp\left\{i\left(kx-\omega t\right)\right\}+c.c.,$$

where Γ≪1 is the amplitude of the perturbation and c.c. represents the complex conjugate. The real number *k* denotes the wave number, and ω is the complex wave velocity expressed as $\omega =\omega_{r}+i\omega_{i}$. Substituting Equation (83) into Equation (79) and considering its linear part, we obtain the following dispersion relation:

(84) $$D\left(\omega ,k\right)=-i\omega +Aik-Bk^{2}+Ck^{4}=0.$$

Substituting the expression of ω into Equation (84), the real and imaginary parts of ω are obtained as

(85) $$\begin{array}{cc} \; & \omega_{r}=Ak,\\ \; & \omega_{i}=Bk^{2}-Ck^{4}, \end{array}$$

In Equation (85), $\omega_{r}$ denotes the oscillation frequency and $\omega_{i}$ is the linear growth rate of the amplitude. When $\omega_{i}=0$ and $\partial^{2}\omega_{i}/\partial k^{2}=0$ [26], we can obtain the expression of the critical Reynolds number

(86) $$\begin{array}{cc} Re_{c}= & \frac{15}{8+48\beta +100\beta^{2}+60\beta^{3}}(-2\Lambda +\frac{2}{3}\cot\theta +\frac{2E}{3{\left(1-d\right)}^{3}}+2\eta -8\Lambda \beta +6\eta \beta +2\beta \cot\theta \\ \; & +\frac{2E\beta }{{\left(1-d\right)}^{3}}-8\Lambda \beta^{2}+4\eta \beta^{2}). \end{array}$$

From Equation (86), it can be concluded that, when we ignore the electric field, odd viscosity, porous medium parameters, and retardation time (i.e., when E=0, η=0, β=0, and Λ=0), the expression in Equation (86) corresponds to the classical critical Reynolds value $Re^{*}=5\cot\theta /4$ of the inclined plane, which is consistent with existing results.

For the linear stability criterion described in Equation (85), we first fix the height d=2 between the two plates and the inclination angle $\theta =45^{\circ }$. By making $\omega_{i}=0$, a neutral stability curve can be obtained, which indicates that the flow is stable when $\omega_{i}<0$, unstable when $\omega_{i}>0$, and neutral and stable when $\omega_{i}=0$. In other words, the region represented by $\omega_{i}\leq 0$ is stable and the region represented by $\omega_{i}>0$ is unstable. When both the electric field and the odd viscosity are neglected, the critical Reynolds number becomes

$$Re_{c}=Re^{*}=\frac{15(\frac{2}{3}\cot\theta +2\beta \cot\theta -2\Lambda -8\beta \Lambda -8\beta^{2}\Lambda )}{\left(8+48\beta +100\beta^{2}+60\beta^{3}\right)}.$$

**Figure 2:**
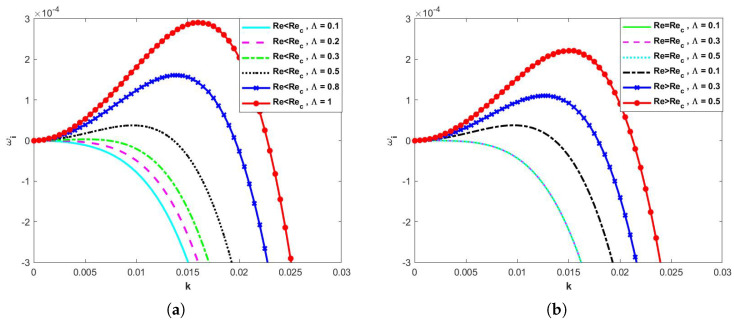
Dispersion relations for long−wave models when d=2, E=0, η=0, β=0.1, $\theta =45^{\circ }$, and Ca=0.0001. (**a**) $\;Re<Re_{c}$ and (**b**) $\;Re\geq Re_{c}$.

**Figure 3:**
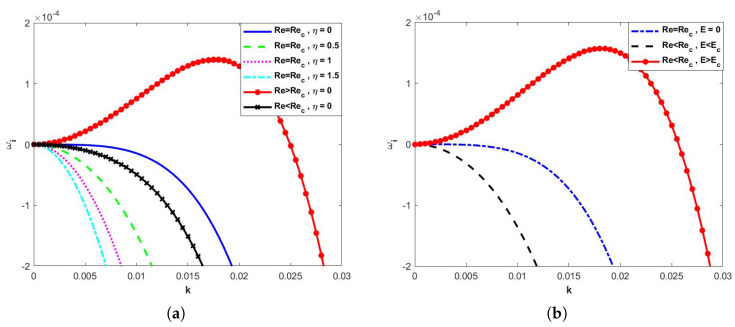
Dispersion relations for long−wave models when d=2, β=0.1, $\theta =45^{\circ }$, Λ=0.1, Ca=0.0003. (**a**)E=0, and (**b**) η=0.

**Figure 4:**
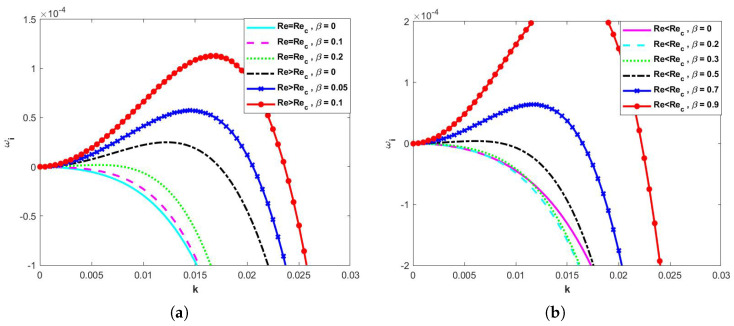
Dispersion relations for long−wave models when d=2, E=0, η=0, Λ=0.1, $\theta =45^{\circ }$, and Ca=0.0003. (**a**) $\;Re\geq Re_{c}$ and (**b**) $\;Re<Re_{c}$.

**Figure 5:**
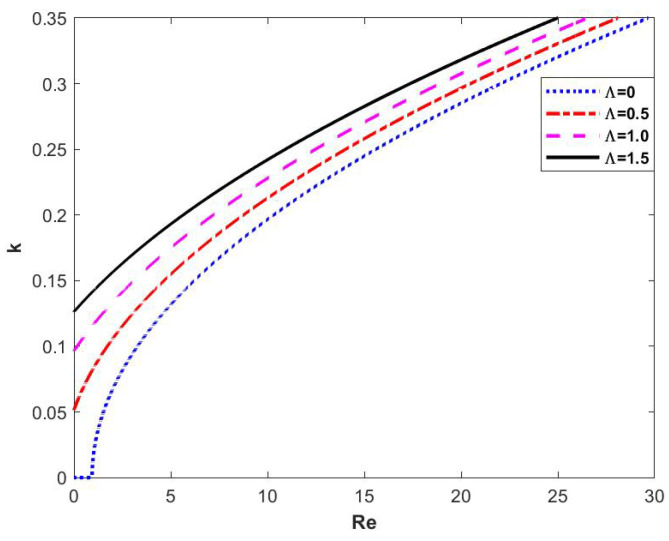
Neutral stability curve when changing Λ at $\theta =45^{\circ }$, d=2, β=0.1, η=0, and E=0.

**Figure 6:**
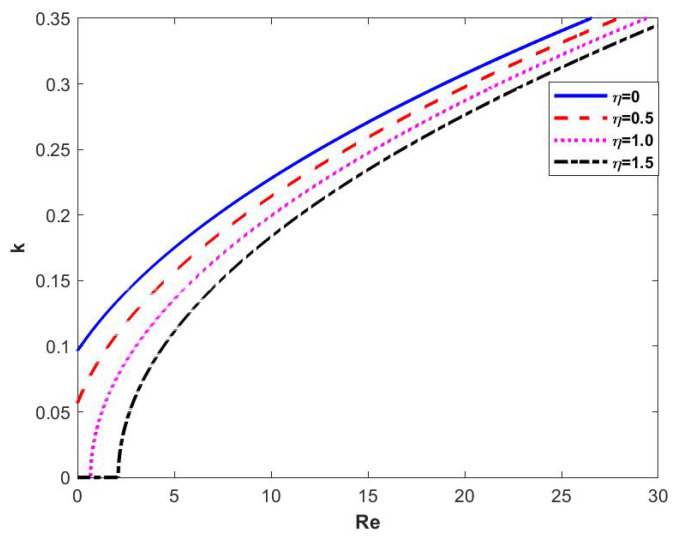
Neutral stability curve when changing η at $\theta =45^{\circ }$, d=2, β=0.1, Λ=1, and E=0.

**Figure 7:**
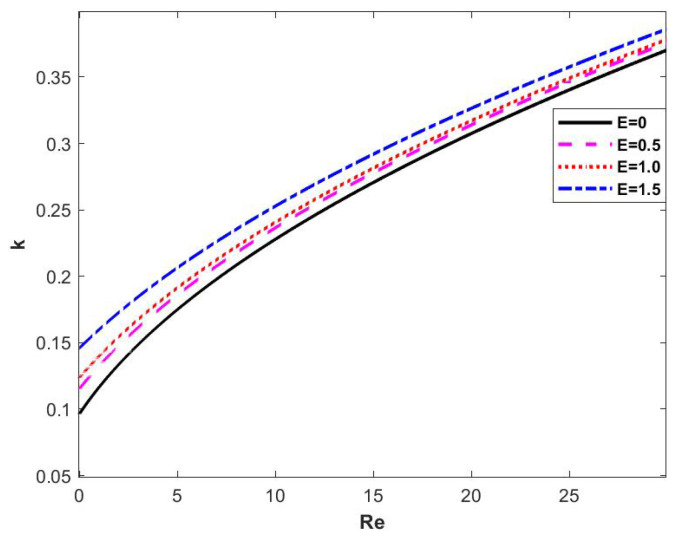
Neutral stability curve when changing *E* at $\theta =45^{\circ }$, d=2, β=0.1, Λ=1, and η=0.

**Figure 8:**
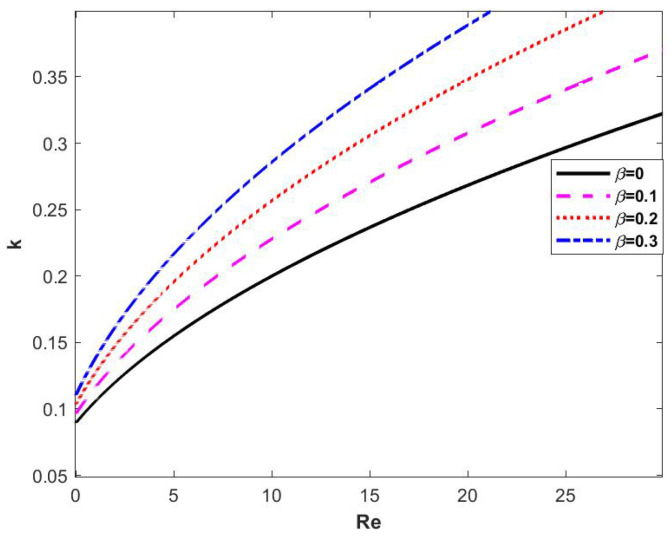
Neutral stability curve when changing β at $\theta =45^{\circ }$, d=2, E=0, Λ=1, and η=0.

Linear stability analysis based on Equations (85) and (86) yields Figure 2, Figure 3, Figure 4, Figure 5, Figure 6, Figure 7 and Figure 8. As shown in Figure 2a,b, we vary the magnitude of the retardation time Λ for the Oldroyd-B fluid, and, from Figure 2a, it can be seen that, when $Re<Re^{*}$, the neutral stability curve keeps shifting upwards as the retardation time Λ continues to increase, i.e., the value of the linear growth rate $\omega_{i}$ gradually increases, and, at this point, the flow becomes unstable, i.e., the retardation time Λ value has a destabilizing effect. As can be seen from Figure 2b, when $Re=Re^{*}$, the value of $\omega_{i}$ is always less than 0 and, no matter how the value of Λ is changed, the difference in the value of $\omega_{i}$ is extremely small, so the three lines are almost coincident, and, therefore, the flow is stable. When $Re>Re^{*}$, the flow becomes unstable as Λ keeps increasing.

When the height between the two fixed plates d=2 and the inclination angle $\theta =45^{\circ }$, neglecting the effect of the electric field, it can be seen from Figure 3a that, when $Re\leq Re^{*}$, as the odd viscosity coefficient η continues to increase, $\omega_{i}$ is always less than 0, i.e., the flow is always stable. When the effect of the odd viscosity is neglected, the critical electrical parameters become

$$E_{c}=\frac{3{\left(1-d\right)}^{3}}{2+6\beta }\left[\frac{Re(8+48\beta +100\beta^{2}+60\beta^{3})}{15}-\frac{2}{3}\left(1+3\beta \right)\cot\theta +2\Lambda +8\beta \Lambda +8\beta^{2}\Lambda \right].$$

From Figure 3b, it can be seen that, when $Re<Re^{*}$, $E<E_{c}$, the flow is stable, and, when $Re<Re^{*}$, $E>E_{c}$, the linear growth rate $\omega_{i}$ increases and then decreases, and the flow becomes unstable.

When both the electric field and the odd viscosity are neglected, varying the magnitude of the porous medium parameter β, it can be seen from the dispersion relation between $\omega_{i}$ and *k* shown in Figure 4 that, in Figure 4a, when $Re=Re^{*}$, $\omega_{i}$ is always less than 0, and therefore the flow is stable, and when $Re>Re^{*}$, the flow becomes unstable as β continues to increase. In Figure 4b, when $Re<Re^{*}$, the flow is stable at β<0.5; when β≥0.5, the flow becomes unstable as $\omega_{i}$ starts to increase and $\omega_{i}>0$.

In Equation (83), by making $\omega_{i}=0$, the neutral stability curve is obtained, which reflects the variation of wave number *k* with the Reynolds number Re. In Figure 5, we first fix the magnitude of other parameters and change the magnitude of the retardation time Λ. The upper left corner of the figure shows the stabilized zone and the lower right corner shows the unstable zone, and it can be seen that the neutral stability curve moves to the upper left corner when Λ keeps on increasing, so that the stable region is decreasing, the unstable region keeps on expanding, and the critical Reynolds number decreases with it. It can be seen that the magnitude of the retardation time Λ has an unsteady effect on the flow of the Oldroyd-B fluid film.

In Figure 6, when the magnitude of the odd viscosity η is varied and the other parameters are fixed, we can see that the critical Reynolds number increases with the increase of the odd viscosity η, the neutral stability curve profile shifts to the right and the value of the wave number *k* decreases. In other words, the odd viscosity has a stabilizing effect on the flow of the film, and the larger η is, the more stable the flow is.

When the other parameters are fixed and the electrical parameter *E* is changed, the neutral stability curves obtained are shown in Figure 7. The four curves in the figure represent the neutral stability curves obtained when different electrical parameters *E* are selected, and we can see that the critical Reynolds number $Re_{c}$ gradually becomes smaller as the electrical parameter *E* increases, and the neutral stability curves are constantly shifted upward to the left, which results in the manifestation of instability in the film flow.

In Figure 8, the four curves represent the neutral stability curves corresponding to different porous medium parameters β, from which it can be seen that the neutral stability curves keep shifting to the left and the critical Reynolds number is getting smaller and smaller with the increasing of β. In other words, the porous medium parameter β has an unsteady effect on the flow of the film.

## 5. Ginzburg–Landau Equation

In order to obtain a nonlinear stability judgment criterion for the system, we can transform the problem. For this purpose, we will use a multiscale approach to extend the perturbation interface $h_{1}\left(x,t\right)$. The basic idea of this approach is to extend the solution of the problem as a function of two or more independent variables, which in turn leads to the derivation of the Ginzburg–Landau equation, by which the stability of the flow can be judged. The detailed derivation of the Ginzburg–Landau equation is given in Appendix A.

After derivation, we can obtain the following equation:

(87) $$\frac{\partial a}{\partial t_{2}}=\left(\varepsilon^{-2}\omega_{i}-J_{2}a^{2}\right)a,$$

(88) $$\frac{\partial \left[b\left(t_{2}\right)t_{2}\right]}{\partial t_{2}}=a^{2}J_{3}.$$

Equation (87) is the Ginzburg–Landau equation, with the second term on the right-hand side being a nonlinear term, which can either moderate or accelerate the exponential growth of the linear perturbation depending on the sign of $\omega_{i}$ and $J_{2}$. If $J_{2}=0$, Equation (87) reduces to the equation given in linear theory. When the right-hand side of Equation (87) is 0, the equilibrium amplitude can be solved as

(89) $$\varepsilon a={\left(\frac{\omega_{i}}{J_{2}}\right)}^{1/2}.$$

**Figure 9:**
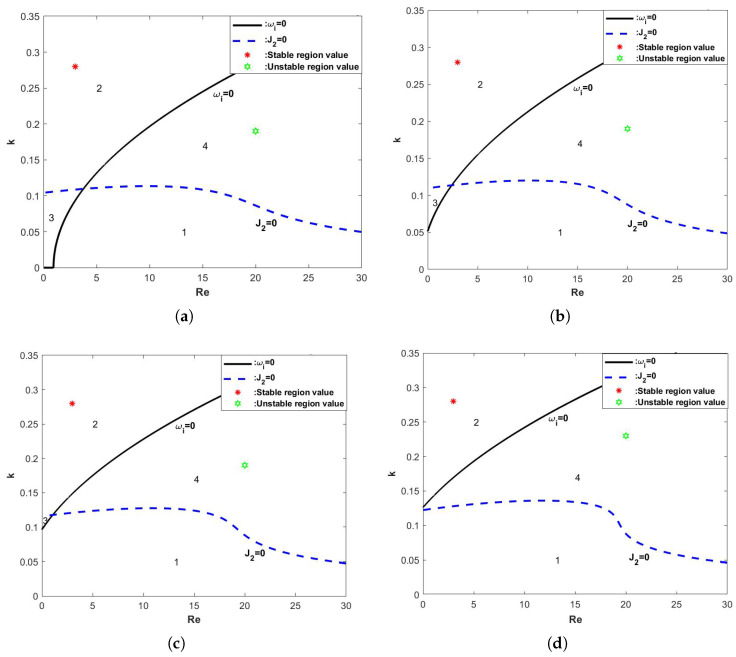
The stability curve wave number *k* varies with Re when the $\theta =45^{\circ }$, d=2, E=0, β=0.1, and η=0. (**a**) Λ=0, (**b**) Λ=0.5, (**c**) Λ=1, and (**d**) Λ=1.5.

**Figure 10:**
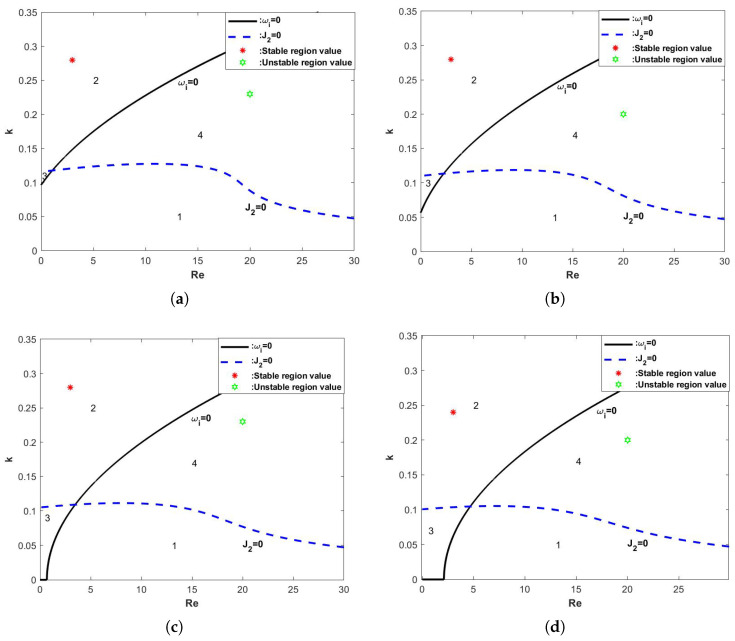
The stability curve wave number *k* varies with Re when the $\theta =45^{\circ }$, d=2, E=0, β=0.1, and Λ=1. (**a**) η=0, (**b**) η=0.5, (**c**) η=1, and (**d**) η=1.5.

**Figure 11:**
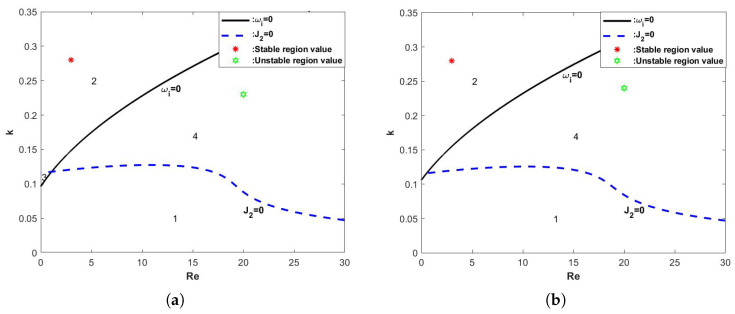

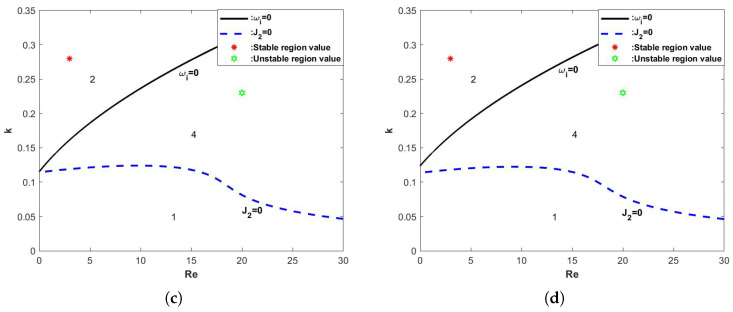
The stability curve wave number *k* varies with Re when the $\theta =45^{\circ }$, d=2, η=0, β=0.1, and Λ=1. (**a**) E=0, (**b**) E=0.5, (**c**) E=1, and (**d**) E=1.5.

**Figure 12:**
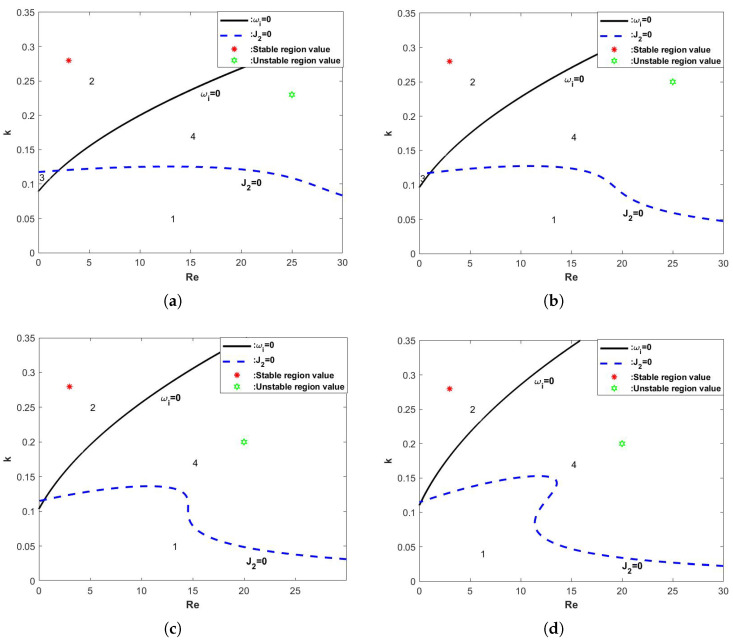
The stability curve wave number *k* varies with Re when the $\theta =45^{\circ }$, d=2, η=0, E=0, and Λ=1. (**a**) β=0, (**b**) β=0.1, (**c**)β=0.2, and (**d**)β=0.3.

When $J_{2}>0$, the saturation of the amplitude is ensured, whereas when $J_{2}<0$, the saturation of the amplitude will not occur, so $J_{2}<0$ will lead to system instability. Based on the sign of $\omega_{i}$ and $J_{2}$, the following four nonlinear regions are defined: supercritical stabilization region 1 ($\omega_{i}>0,J_{2}>0$), subcritical instability region 2 ($\omega_{i}<0,J_{2}<0$), unconditional stabilization region 3 ($\omega_{i}<0,J_{2}>0$), and the explosive state region 4 ($\omega_{i}>0,J_{2}<0$) [39]. Therefore, nonlinear stability analysis based on Equations (85) and (103) yields Figure 9, Figure 10, Figure 11 and Figure 12.

As can be seen in Figure 9, when the electric field and odd viscosity are neglected, the critical Reynolds number $Re_{c}$ decreases with increasing retardation time Λ for the Oldroyd-B fluid, as well as region 2 and region 3, but region 1 and region 4 increase. This shows that the retardation time Λ has an unsteady effect on the flow of the film.

When the odd viscosity coefficient η increases, so that $\omega_{i}=0$ and $J_{2}=0$, the resulting graph is shown in Figure 10, which shows that the critical Reynolds number $Re_{c}$ is increasing as η is increasing, and regions 2 and 3 are increasing, and regions 1 and 4 are decreasing. In other words, it is the odd viscosity coefficient that plays a stabilizing role in the flow of the liquid film.

Figure 11 demonstrates the stability curves determined by the wave number *k* and the Reynolds number Re for different electric parameters *E*. It can be seen that, when the electric field strength *E* keeps increasing, the regions 2 and 3 gradually decrease, the regions 1 and 4 gradually increase, and the critical Reynolds number keeps decreasing. In other words, the addition of an electric field makes the fluid more unstable.

When the porous medium parameter β is changed, the stability curves obtained are shown in Figure 12, and it can be seen that the critical Reynolds number decreases with the increase of β. Regions 2 and 3 decrease with the increase of β, while regions 1 and 4 gradually increase, which shows that the porous medium parameterization plays an unsteady role in the flow of viscoelastic liquid film.

## 6. Numerical Analysis of Nonlinear Evolution Equations

The solution of nonlinear evolution equations has been an important topic in the study of flow stability. In order to solve this class of equations, we use the Fourier transform method, which discretizes the spatial variables into a series of wave numbers, so that the problem can be reduced to solving a set of ordinary differential equations, and, thus, the fast Fourier transform method can be used to calculate these wave numbers. Finally, we can obtain the numerical solution of the nonlinear evolution equations.

In this study, we used the perturbation of the most unstable wave number $k_{m}$ as an initial condition with the expression $h=1+0.05\cos k_{m}x$. This is because the mode corresponding to the most unstable wave number $k_{m}$ gradually strengthens during the evolution process, which leads to a change in the behavior of the whole system. The least stable wave number $k_{m}$ can be obtained by solving $d\omega_{r}/dk=0$.

In Equation (78) of the nonlinear evolution equation, we conduct the Fourier transform of the variable *x* to obtain

$$\begin{array}{cc} \frac{\partial \hat{h}}{\partial t}= & -ik\cdot F\{\frac{2}{3}F^{-1}{\left[\hat{h}\right]}^{3}+2\beta F^{-1}{\left[\hat{h}\right]}^{2}+\frac{8}{15}ReF^{-1}{\left[\hat{h}\right]}^{6}F^{-1}\left[ik\hat{h}\right]+\frac{16}{5}Re\beta F^{-1}{\left[\hat{h}\right]}^{5}F^{-1}\left[ik\hat{h}\right]\\ \; & +\left(\frac{20}{3}Re\beta^{2}+2\Lambda \right)F^{-1}{\left[\hat{h}\right]}^{4}F^{-1}\left[ik\hat{h}\right]-\frac{2}{3}(-12\beta \Lambda +\cot\theta +\frac{E}{{\left(F^{-1}\left[\hat{h}\right]-d\right)}^{3}}+3\eta \\ \; & -6Re\beta^{3})F^{-1}{\left[\hat{h}\right]}^{3}F^{-1}\left[ik\hat{h}\right]-\left(-8\beta^{2}\Lambda +6\eta \beta +2\beta \cot\theta +\frac{2E\beta }{{\left(F^{-1}\left[\hat{h}\right]-d\right)}^{3}}\right)\\ \; & \times F^{-1}{\left[\hat{h}\right]}^{2}F^{-1}\left[ik\hat{h}\right]-4\eta \beta^{2}F^{-1}\left[\hat{h}\right]F^{-1}\left[ik\hat{h}\right]+\frac{1}{3Ca}F^{-1}{\left[\hat{h}\right]}^{3}F^{-1}\left[{\left(ik\right)}^{3}\hat{h}\right]\\ \; & +\frac{\beta }{Ca}F^{-1}{\left[\hat{h}\right]}^{2}F^{-1}\left[{\left(ik\right)}^{3}\hat{h}\right]\} \end{array}$$

**Figure 13:**
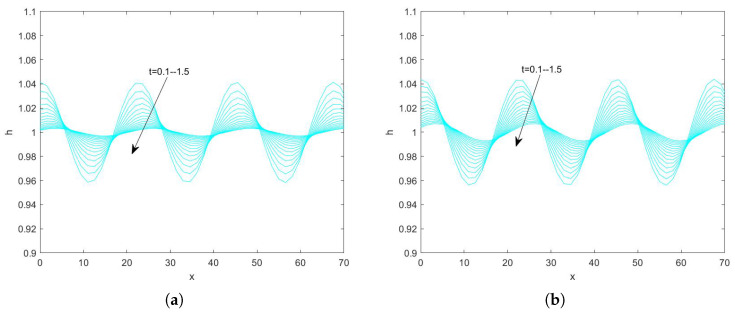
The short−time evolution of the free surface when Re=3, $\theta =45^{\circ }$, d=2, Ca=0.0003, η=0.5, β=0.1, and E=0.5. (**a**) Λ=0.5 and (**b**) Λ=3.

**Figure 14:**
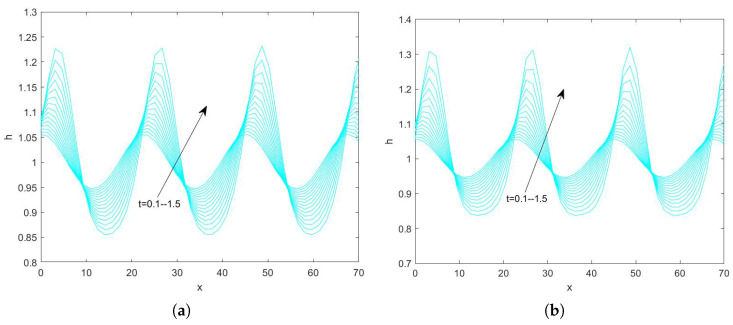
The short−time evolution of the free surface when Re=20, $\theta =45^{\circ }$, d=2, Ca=0.001, η=0.5, β=0.1, and E=0.5. (**a**) Λ=0.5 and (**b**) Λ=1.

**Figure 15:**
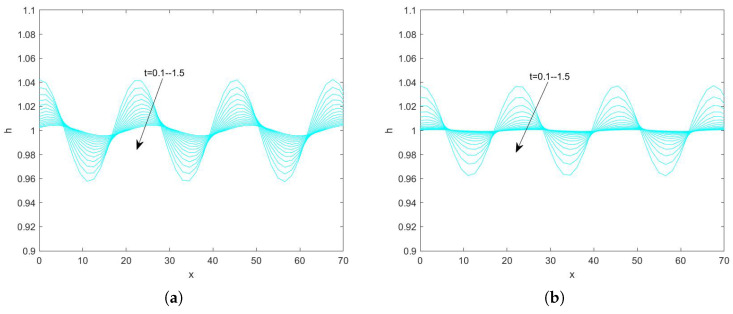
The short−time evolution of the free surface when Re=3, $\theta =45^{\circ }$, d=2, Ca=0.0003, Λ=0.5, β=0.1, and E=0.5. (**a**) η=0 and (**b**) η=6.

**Figure 16:**
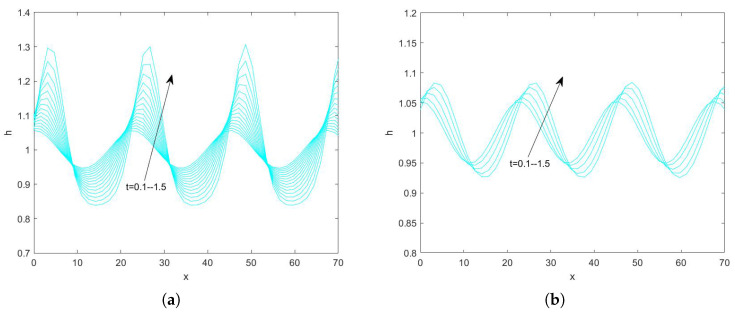
The short−time evolution of the free surface when Re=20, $\theta =45^{\circ }$, d=2, Ca=0.001, Λ=0.5, β=0.1, and E=0.5. (**a**) η=0 and (**b**) η=3.

**Figure 17:**
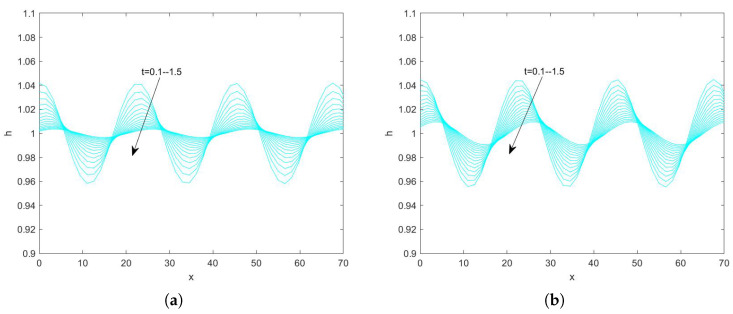
The short−time evolution of the free surface when Re=3, $\theta =45^{\circ }$, d=2, Ca=0.0003, Λ=0.5, β=0.1, and η=0.5. (**a**) E=0 and (**b**) E=10.

**Figure 18:**
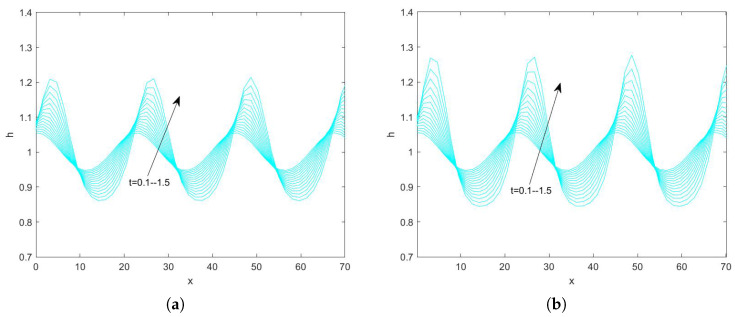
The short−time evolution of the free surface when Re=20, $\theta =45^{\circ }$, d=2, Ca=0.001, Λ=0.5, β=0.1, and η=0.5. (**a**) E=0 and (**b**) E=1.5.

**Figure 19:**
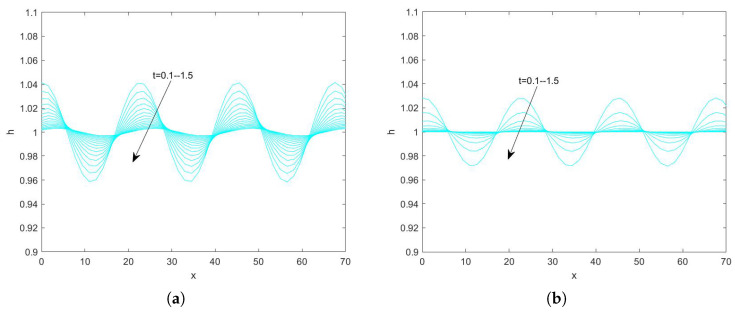
The short−time evolution of the free surface when Re=3, $\theta =45^{\circ }$, d=2, Ca=0.0003, Λ=0.5, E=0.5, and η=0.5. (**a**) β=0.1 and (**b**) β=0.3.

**Figure 20:**
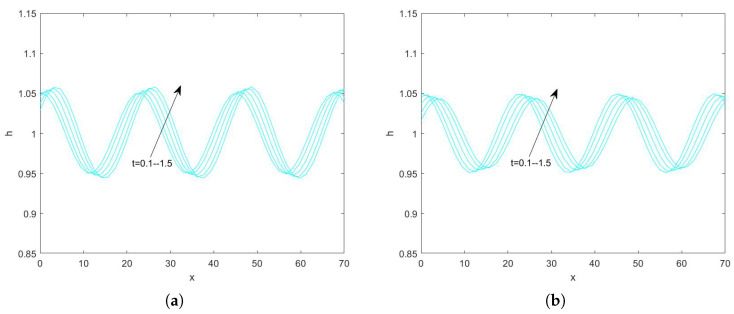

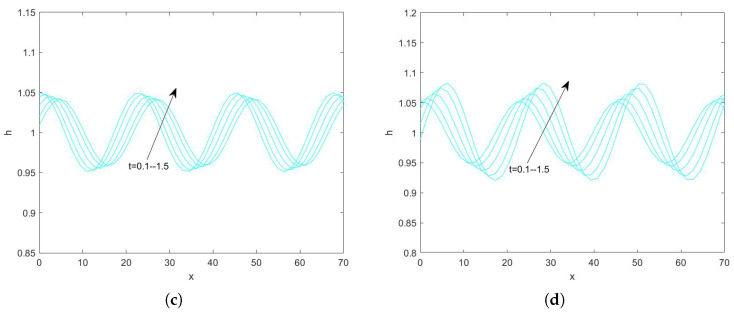
The short−time evolution of the free surface when Re=10, $\theta =45^{\circ }$, d=2, Ca=0.001, Λ=0.5, E=0.5, and η=0.5. (**a**) β=0.1 and (**b**) β=0.2, (**c**) β=0.3, and (**d**) β=0.5.

In the use of the Fourier transform for solving nonlinear evolution equations, we assume that the wave on which it acts exists on a range with period 2π. First, we convert the problem to an ordinary differential equation problem. Second, we perform the iterative solution using the “ode45” function in MATLAB(R2021a), which is specialized in solving ordinary differential equations. Finally, the solution obtained on the period domain is mapped to a solution that applies to an infinitely long interval. By plotting the pattern, the state of the liquid film flow is determined and the effect of each parameter on the flow stability is examined. Numerical analysis of Equation (78) using the Fourier spectrum method yields Figure 13, Figure 14, Figure 15, Figure 16, Figure 17, Figure 18, Figure 19 and Figure 20.

Figure 13 illustrates the evolution of the free surface of the film obtained by fixing the values of the other parameters and varying the retardation time Λ when Re=3. From the above figure, it can be seen that, when the time span is 0–1.5, the amplitude of the free surface becomes gradually smaller with the increase of time, which indicates that the perturbation is gradually weakened with the increase of time, and the flow of the film gradually becomes stable. Figure 14 shows the interfacial evolution obtained by varying the retardation time Λ when Re=20, from which it can be seen that, when Λ is increased from 0.5 to 1.5, the perturbation is gradually strengthened with the increase of time, and the amplitude of the free surface is increased from 1.23 to 1.32, which shows that the flow becomes more and more unstable. In conclusion, the flow of the liquid film becomes unstable as the retardation time increases.

In Figure 15 and Figure 16, we investigate the interface evolution plots obtained by varying the magnitude of the odd viscosity coefficient η for Reynolds numbers 3 and 20. In Figure 15, fixing the magnitude of other parameters, the peak of the interface is about 1.043 when η=0, and about 1.038 when η=6. And the interface gradually becomes smooth with the increase of time. In Figure 16, when η=0, the peak value of the interface is 1.3, and, when η=3, the peak value becomes about 1.082. In conclusion, both Figure 15 and Figure 16 show that the peak value of the free surface gradually becomes smaller as the odd viscosity coefficient increases and the flow becomes stable.

Figure 17 shows the evolution of the interface obtained by fixing the magnitude of the other parameters and varying the electrical parameter *E* when Re=3. It can be seen that the amplitude of the interface gradually decreases with the increase of time, indicating that the perturbation is gradually weakened with the increase of time and the film flow is gradually stabilized. The peak value of the interface is 1.04 when E=0, and 1.043 when E=10. Figure 18 shows the evolution of the interface when Re=20, and it can be seen that the peak value of the interface increases with the increase of the electrical parameter, and the perturbation increases with time, and the flow becomes unstable. In conclusion, both Figure 17 and Figure 18 show that the peak value of the free surface increases with the increase of the electrical parameter and the liquid film flow becomes unstable.

Figure 19 and Figure 20 depict the interfacial evolution corresponding to different porous media parameters for Reynolds numbers 3 and 10, respectively. In Figure 19, the amplitude of the free surface decreases with time; thus, the perturbation decreases and the flow stabilizes. It is worth mentioning that, at β=0.3, the flow in the film turns into a standing wave form with increasing time. In Figure 20, the peak at the free surface is about 1.053 when β=0.1, the peak at the interface decreases to 1.051 when β=0.2, the peak is about 1.05, when β=0.3, and the peak at the free surface rises to about 1.083 when β=0.5, which suggests that the flow becomes more and more stable with the increase of the porous medium parameter, the perturbation first weakens and then strengthens, and, when β<0.5, the flow stabilizes with the increase of β. When β≥0.5, the flow becomes unstable with increasing β. This result is consistent with the results obtained from the linear stability analysis.

## 7. Conclusions

In this paper, we investigate the effect of odd viscosity on the stability of a thin film of Oldroyd-B viscoelastic fluid flowing along a porous inclined surface in the presence of a normal electric field. We first assume that the flow of the film on the porous inclined plane satisfies the Beavers–Joseph slip boundary condition, and then the equations of motion and boundary conditions are processed by using the long-wave approximation and the asymptotic expansion method to derive the nonlinear evolution equation of the film flow, followed by a series of stability analyses.

By neglecting the nonlinear terms in the nonlinear evolution equations, dispersion relations and neutral stability curves used in the linear stability analysis were obtained. Several conclusions were drawn: first, the flow of the film becomes unstable as the delay time of the Oldroyd-B fluid increases; second, the odd viscosity plays a stabilizing role in the film flow; third, an increase in the electric field parameter destabilizes the flow of the liquid film; and, finally, an increase in the parameters of the porous medium leads to flow instability.

In the nonlinear stability analysis, we analyzed the nonlinear evolution equations by using the multiscale method and obtained the Ginzburg–Landau equation, from which we obtained the nonlinear stability criterion. The results show that, as the odd viscosity increases, the unconditional stability region 2 and subcritical instability region 3 will increase, while the supercritical stability region 1 and the explosion region 4 will decrease, which indicates that the odd viscosity plays a stabilizing role for the liquid film flow. On the contrary, with the increase of fluid delay time, electrical parameter, and porous medium parameter, the unconditionally stable region 2 and subcritical unstable region 3 will keep shrinking, while the supercritical stable region 1 and explosive region 4 will keep increasing, which indicates that the addition of fluid delay time, electrical parameter, and porous slope permeability destabilizes the liquid film flow.

Finally, we numerically simulate the nonlinear evolution equations of the liquid film flow using the Fourier spectral method, and the results obtained are consistent with those obtained from the linear stability analysis, i.e., in the stable region, fixing the magnitude of the delay time, the singular viscous coefficient, the electrical parameter, and the porous slanting parameter of the fluid, the perturbations are all decreasing with the increase of the time, and the amplitude of the flow is also decreasing, and the flow is stabilized gradually. On the contrary, in the unstable region, also fixing the magnitude of these parameters, we find that the amplitude of the perturbations gradually increases with time and the flow is destabilized, which is consistent with the results obtained from the linear stability analysis.

For the study of the stability of Oldroyd-B fluid films with odd viscosity flowing on porous substrates, only the theoretical part of the study is presented in this paper, and we believe that it can contribute to practical applications. For example, in the field of petroleum engineering, crude oil in reservoirs often exhibits non-Newtonian fluid properties, similar to Oldroyd-B fluids with odd viscosity. The study of its flow stability in porous substrates helps to understand the seepage pattern of crude oil in the reservoir. By analyzing the stability, reservoir recovery schemes can be optimized, such as determining reasonable injection and extraction parameters and improving the efficiency of oil drive, thus maximizing reservoir recovery and increasing oil production [47]. In the field of biomedical engineering, many fluids in living organisms, such as blood and joint fluids, have non-Newtonian fluid properties and flow in porous biological tissues (e.g., blood vessel walls, cartilage, etc.). The study of the flow stability of Oldroyd-B fluids with odd viscosity on porous substrates can help to establish more accurate biofluid dynamics models to simulate the fluid flow in organisms, and provide theoretical support for the study of the pathogenesis of cardiovascular diseases, joint-type disorders, and so on [48].

## Data Availability

The data that support the findings of this study are available from the corresponding author upon reasonable request.

## Appendix A

The Ginzburg–Landau equation is derived as follows:

Let us introduce

(A1) $$\begin{array}{cc} \; & x_{1}=\varepsilon x,\\ \; & t_{1}=\varepsilon t,t_{2}=\varepsilon^{2}t.\cdots \end{array}$$

Then, the disturbed film thickness $h_{1}$ can be expressed as [39]

(A2) $$h_{1}\left(x,x_{1},\cdots ,t,t_{1},t_{2}\right)=\varepsilon \eta_{1}+\varepsilon^{2}\eta_{2}+\varepsilon^{3}\eta_{3}+\cdots .$$

Therefore, defining *t* and *x* as variables appropriate for fast variation, $x_{1}$, $t_{1}$, and $t_{2}$ are slow variables. The differential operators can now be expressed as the derivative expansion

(A3) $$\begin{array}{cc} \; & \frac{\partial }{\partial t}=\frac{\partial }{\partial t}+\varepsilon \frac{\partial }{\partial t_{1}}+\varepsilon^{2}\frac{\partial }{\partial t_{2}},\\ \; & \frac{\partial }{\partial x}=\frac{\partial }{\partial x}+\varepsilon \frac{\partial }{\partial x_{1}}. \end{array}$$

The substitution of Equations (A2) and (A3) into Equation (79) gives us

(A4) $$\begin{array}{cc} \left(L_{0}+\varepsilon L_{1}+\varepsilon^{2}L_{2}+\cdots \right) & \left(\varepsilon \eta_{1}+\varepsilon^{2}\eta_{2}+\varepsilon^{3}\eta_{3}+\cdots \right)=-\varepsilon^{2}N_{2}-\varepsilon^{3}N_{3}-\cdots \end{array}$$

The following expressions for the operators $L_{0}$, $L_{1}$, $L_{2}$, $N_{2}$, and $N_{3}$ in Equation (A4) are given below, where *L* stands for the linear operator and *N* stands for the nonlinear operator:

$$\begin{array}{cc} L_{0}= & \frac{\partial }{\partial t}+A\frac{\partial }{\partial x}+B\frac{\partial^{2}}{\partial x^{2}}+C\frac{\partial^{4}}{\partial x^{4}},\\ L_{1}= & \frac{\partial }{\partial t_{1}}+A\frac{\partial }{\partial x_{1}}+2B\frac{\partial^{2}}{\partial x\partial x_{2}}+4C\frac{\partial^{4}}{\partial x^{3}\partial x_{1}},\\ L_{2}= & \frac{\partial }{\partial t_{2}}+B\frac{\partial^{2}}{\partial x_{1}^{2}}+6C\frac{\partial^{4}}{\partial x^{2}\partial x_{1}^{2}},\\ N_{2}= & A^{\prime }\eta_{1}\frac{\partial \eta_{1}}{\partial x}+B^{\prime }\left[\eta_{1}\frac{\partial^{2}\eta_{1}}{\partial x^{2}}+{\left(\frac{\partial \eta_{1}}{\partial x}\right)}^{2}\right]+C^{\prime }\left[\eta_{1}\frac{\partial^{4}\eta_{1}}{\partial x^{4}}+\frac{\partial \eta_{1}}{\partial x}\frac{\partial^{3}\eta_{1}}{\partial x^{3}}\right],\\ N_{3}= & A^{\prime }\left[\eta_{1}\left(\frac{\partial \eta_{2}}{\partial x}+\frac{\partial \eta_{1}}{\partial x_{1}}\right)+\eta_{2}\frac{\partial \eta_{1}}{\partial x}\right]+B^{\prime }[\eta_{1}\left(\frac{\partial^{2}\eta_{2}}{\partial x^{2}}+2\frac{\partial^{2}\eta_{1}}{\partial x\partial x_{1}}\right)+\eta_{2}\frac{\partial^{2}\eta_{1}}{\partial x^{2}}+2\frac{\partial \eta_{1}}{\partial x_{1}}(\frac{\partial \eta_{2}}{\partial x_{1}}\\ \; & +\frac{\partial \eta_{1}}{\partial x_{1}})]+C^{\prime }[\eta_{1}\left(\frac{\partial^{4}\eta_{2}}{\partial x^{4}}+4\frac{\partial^{4}\eta_{1}}{\partial x^{3}\partial x_{1}}\right)+\eta_{2}\frac{\partial^{4}\eta_{1}}{\partial x^{4}}+\frac{\partial \eta_{1}}{\partial x}\left(\frac{\partial^{3}\eta_{2}}{\partial x^{3}}+3\frac{\partial^{3}\eta_{1}}{\partial x^{2}\partial x_{1}}\right)+\frac{\partial^{3}\eta_{1}}{\partial x^{3}}(\frac{\partial \eta_{2}}{\partial x}\\ \; & +\frac{\partial \eta_{1}}{\partial x_{1}})]+\frac{1}{2}A^{\prime \prime }\eta_{1}^{2}\frac{\partial \eta_{1}}{\partial x}+B^{\prime \prime }\left[\frac{1}{2}\eta_{1}^{2}\frac{\partial^{2}\eta_{1}}{\partial x^{2}}+\eta_{1}{\left(\frac{\partial \eta_{1}}{\partial x}\right)}^{2}\right]+C^{\prime \prime }\left(\frac{1}{2}\eta_{1}^{2}\frac{\partial^{4}\eta_{1}}{\partial x^{4}}+\eta_{1}\frac{\partial \eta_{1}}{\partial x}\frac{\partial^{3}\eta_{1}}{\partial x^{3}}\right). \end{array}$$

And then, comparing the powers of ε on both sides of Equation (A4), one obtains the following three orders in ε:

(A5) $$\begin{array}{cc} \; & \varepsilon^{1}:\;L_{0}\eta_{1}=0,\\ \; & \varepsilon^{2}:\;L_{0}\eta_{2}=-L_{1}\eta_{1}-N_{2},\\ \; & \varepsilon^{3}:\;L_{0}\eta_{3}=-L_{1}\eta_{2}-L_{2}\eta_{1}-N_{3} \end{array}$$

According to the first-order problem O(ε),

(A6) $$L_{0}\eta_{1}=0,$$

We assume that the equation has a solution of the following form:

(A7) $$\eta_{1}=\Gamma \left(x_{1},t_{1},t_{2}\right)\exp i\left(kx-\omega_{r}t\right)+c.c.,$$

In Equation (A7), $\Gamma (x_{1},\;t_{1},\;t_{2})$ denotes the complex amplitude. This solution is obtained from Equation (83) in the linear stability analysis, but since $\omega_{i}$ is of order O(ε) in the vicinity of the neutral stability curve and the function $\exp(\omega_{i}t)$ is slow and can be absorbed by $\Gamma (x_{1},t_{1},t_{2})$, $\omega_{r}$ is used instead of ω. For the second-order equation $O(\varepsilon^{2})$,

(A8) $$L_{0}\eta_{2}=-L_{1}\eta_{1}-N_{2},$$

Substituting Equation (A7) into Equation (A8) yields

(A9) $$\begin{array}{cc} L_{0}\eta_{2}= & -i\left[\frac{\partial D_{1}\left(\omega_{r},k\right)}{\partial \omega_{r}}\frac{\partial \Gamma }{\partial t_{1}}-\frac{\partial D_{1}\left(\omega_{r},k\right)}{\partial k}\frac{\partial \Gamma }{\partial x_{1}}\right]e^{i\left(kx-\omega_{r}t\right)}-\Omega \Gamma^{2}e^{2i\left(kx-\omega_{r}t\right)}+c.c., \end{array}$$

where

$$\begin{array}{cc} \; & D_{1}\left(\omega_{r},k\right)=-i\omega_{r}+Aik-Bk^{2}+Ck^{4},\\ \; & \Omega =iA^{\prime }k-2B^{\prime }k^{2}+2C^{\prime }k^{4}. \end{array}$$

By eliminating the long term generated by the first term on the right-hand side of Equation (A9), the solution of Equation (A9) can be expressed as

(A10) $$\eta_{2}=-\frac{\Omega \Gamma^{2}e^{2i\left(kx-\omega_{r}t\right)}}{D_{1}\left(2\omega_{r},2k\right)}+c.c..$$

Let us introduce a coordinate transformation

(A11) $$\xi =(x_{1}-V_{g}t_{1}),$$

where $V_{g}=\left(-\frac{\partial D_{1}}{\partial k}/\frac{\partial D_{1}}{\partial \omega_{r}}\right)$, and, using the solvability condition of the third-order equation, we obtain

(A12) $$\frac{\partial \Gamma }{\partial t_{2}}+J_{1}\frac{\partial^{2}\Gamma }{\partial \xi^{2}}-\varepsilon^{-2}\omega_{i}\Gamma +\left(J_{2}+iJ_{3}\right){\left|\Gamma \right|}^{2}\Gamma =0,$$

The coefficients $J_{1}$, $J_{2}$, and $J_{3}$ in Equation (A12) are expressed as

$$\begin{array}{cc} J_{1}= & B-6Ck^{2},\\ J_{2}= & \frac{1}{2}\left(-B^{\prime \prime }k^{2}+C^{\prime \prime }k^{4}\right)+\left[\frac{{\left(A^{\prime }\right)}^{2}k^{2}-2\left(B^{\prime }k^{2}-7C^{\prime }k^{4}\right)\left(B^{\prime }k^{2}-C^{\prime }k^{4}\right)}{16Ck^{4}-4Bk^{2}}\right],\\ J_{3}= & \frac{1}{2}A^{\prime \prime }k+\left[\frac{A^{\prime }k\left(B^{\prime }k^{2}-7C^{\prime }k^{4}\right)+2A^{\prime }k\left(B^{\prime }k^{2}-C^{\prime }k^{4}\right)}{16Ck^{4}-4Bk^{2}}\right]. \end{array}$$

When the diffusion effect $J_{1}\partial^{2}\Gamma /\partial \xi^{2}$ in Equation (A12) is neglected, one obtains

(A13) $$\frac{\partial \Gamma }{\partial t_{2}}-\varepsilon^{-2}\omega_{i}\Gamma +\left(J_{2}+iJ_{3}\right){\left|\Gamma \right|}^{2}\Gamma =0.$$

We assume that the solution to Equation(A13) is of the form

(A14) $$\Gamma =a\exp\left[-ib\left(t_{2}\right)t_{2}\right],$$

Substituting Equation(A14) into Equation(A13) yields

(A15) $$\frac{\partial a}{\partial t_{2}}=\left(\varepsilon^{-2}\omega_{i}-J_{2}a^{2}\right)a,$$

(A16) $$\frac{\partial \left[b\left(t_{2}\right)t_{2}\right]}{\partial t_{2}}=a^{2}J_{3}.$$

Equation (A15) is the Ginzburg–Landau equation, with the second term on the right-hand side being a nonlinear term, which can either moderate or accelerate the exponential growth of the linear perturbation depending on the sign of $\omega_{i}$ and $J_{2}$. If $J_{2}=0$, Equation(A15) reduces to the equation given in linear theory.
